# Supplementary material for: Continuous structure modification of metal-organic framework glasses via halide salts
Source: Nat Commun. 2025 Jul 30;16:7001. doi: 10.1038/s41467-025-62143-9 (PMC12311036; doi:10.1038/s41467-025-62143-9)
Supplement: Supplementary file 1 — Supplementary Information [file 41467_2025_62143_MOESM1_ESM.pdf]

Supplementary Information

for

Continuous structure modification of metal-organic framework glasses via halide salts

Fengming Cao[1,†], Søren S. Sørensen[1,†,], Anders K. R. Christensen[1], Samraj Mollick[1], Xuan Ge[1], Daming Sun[1], Anders B. Nielsen[2,3], Niels Chr. Nielsen[2,3], Nina Lock[3,4], Ronghui Lu[3,4], Rebekka Klemmt[3,5], Peter K. Kristensen[6], Lars R. Jensen[6], Francesco Dallari[7], Jacopo Baglioni[7], Giulio Monaco[7], Martin A. Karlsen[8], Volodymyr Baran[8], Morten M. Smedskjaer[1,*]*

[1]Department of Chemistry and Bioscience, Aalborg University, DK-9220 Aalborg, Denmark
[2]Department of Chemistry, Aarhus University, Aarhus DK-8000, Denmark
[3]Interdisciplinary Nanoscience Center (iNANO), Aarhus University, DK-8000 Aarhus, Denmark
[4]Department of Biological and Chemical Engineering, Aarhus University, DK-8200 Aarhus N, Denmark
[5]iMAT Aarhus University Centre for Integrated Materials Research, Aarhus University, DK-8000 Aarhus, Denmark
[6]Department of Materials and Production, Aalborg University, Aalborg DK-9220, Denmark
[7]University of Padova, Department of Physics and Astronomy `Galileo Galilei', Via F. Marzolo 8, 35131 Padova, Italy
[8]Deutsches-Elektronen Synchrotron (DESY) Notkestraße 85, 22607, Hamburg, Germany

† These authors contributed equally

* Corresponding Authors.

Emails: Søren S. Sørensen soe@bio.aau.dk; Morten M. Smedskjaer mos@bio.aau.dk

Supplementary Text

Linker evaporation during heating

We have found that the glass transitions of the modified ZIFs are highly reversible upon repeated cooling-heating cycles through the glass transition region, but we have also observed that upon prolonged high-temperature treatment (>300 °C), the T_g of the modified ZIF glasses increases by ~30 °C. For example, this is shown for ZIF-62-bImCl (R=1.0) in Supplementary Fig. 7. This observation is likely enhanced by the large surface-to-volume ratio of the fast-scanning calorimetry sample (microgram range). As described below, we ascribe the increase in T_g to structural changes related to evaporation of organic linkers and repolymerization by introducing bridging halide ions.

First, when preparing the modified ZIF glasses, we employed a setup using glass tubes inserted into a heated aluminum block with the ZIF-modifier sample in the bottom. Upon heating, we observed condensation of a white powder in the colder top part of the tube (Supplementary Fig. 41a). We collected this powder from ZIF-4 and ZIF-62 samples mixed with H_2bImCl (R=1.0) and FT-IR spectroscopy analysis reveals (Supplementary Figs. 41b-g) that the powder is a mixture of imidazole and benzimidazole. Considering that ZIF-4 and H_2bImCl consist of imidazolate and benzimidazolate linkers, respectively, this observation points towards a partial decoordination of the Im from the framework and mixed recoordination with the linker from the added modifier, but also to the removal of excess Im and bIm from the sample. This is further supported by the mass loss observed in fast scanning calorimetry (Supplementary Fig. 7), which, based on the heat flow signal, is found to be ~20% after the described heating procedure to 450 °C.

Structural analysis by FTIR spectroscopy

The FTIR spectra in Supplementary Fig. 15 demonstrate that most of the bands observed in the ZIF-62, ZIF-4, and ZIF-8 crystals are also present in the modified glassy samples, indicating that benzimidazolium chloride does not disrupt many of the structural moieties of ZIF-62, ZIF-4, and ZIF-8, i.e., those belonging to the organic linkers. For the ZIF-8 crystals, there are three characteristic peaks at 1580 cm^{-1} (C=N stretch), 1145 cm^{-1} and 995 cm^{-1} (C−N stretch in the imidazole ring)[2]. These peaks are slightly blue-shifted in the glass samples co-melted with

benzimidazolium chloride modifier. The intense peaks at 1457 and 1382 cm^{-1} correspond to the entire ring stretching. Several spectral bands seen in the range of 1350 to 900 cm^{-1} can be ascribed to the in-plane bending of the ring and the peaks at 760 and 690 cm^{-1} are associated with aromatic sp^2 C-H bending. After mixing and melting ZIF-8 with different amounts of H_2bImCl, the intensities of these peaks are weakened. However, they still exist, which indicates that the metal ion and the ligand remain connected in the modified ZIF glasses.

Compositional analyses using EDX

The sum spectra of the EDX maps (Supplementary Fig. 9) show distinct peaks for C, N, Zn, Cl, and Pt (from coating), confirming the presence of these elements and the absence of other elemental contributions in large quantity. The chemical compositions obtained from the sum spectra are summarized in Supplementary Table 3, but we note that overlaps of nitrogen and carbon signals with the coating and other trace elements make quantification of accurate wt% values difficult.

Supplementary Table 1

Supplementary Table 1. Glass transition temperatures of modified ZIF glasses based on fast scanning calorimetry with a heating rate of 500 K s^{-1}. The transitions were recorded as the onset temperature of the transition.

Composition	Glass transition temperature (°C)
ZIF-62-bImCl R=1	141
ZIF-62-bImCl R=0.5	192
ZIF-62-bImCl R=0.25	261
ZIF-4-bImCl R=1	141
ZIF-4-bImCl R=0.5	211
ZIF-8-bImCl R=1	117
ZIF-8-bImCl R=0.25	215
ZIF-62-bImBr R=1	129
ZIF-62-bImI R=1	138

Supplementary Table 2

Supplementary Table 2. Peak assignments of the Raman modes for crystalline ZIF-4[3]*.

Frequency (cm^{-1})	Assignments
619	In-plane bending vibration of benzimidazole ring
643	Out of plane ring puckering (Im), lattice vib
839	δ_w (C2-H, in-sync C4-H, C5-H)
955 & 977	Im ring vibration
1091	δ_s (C4-H, C5-H) + δ (N3-H)
1111	δ (C2-H, N3-H) + ν (C5-N1) + ν (C4-N3)
1182	Im ring breathing + δr (C2-H, C5-H, N3-H)
1287	r (C2−H, C4−H, C5−H, N3−H) + ν (N3−C2−N1)
1323 & 1345	δ_w (C4-H, C5-H) + ν_{asym} (Im)
1495	ν_{asym} (N1-C2-N3) + δ_r (C2-H, C4-H, C5-H)

*ν = stretching mode, asym = asymmetric, δ = bending mode, r = rocking, t = twisting, w = wagging, s = scissoring, oop = out-of-plane.

Supplementary Table 3

Supplementary Table 3. Elemental composition of the ZIFs in wt% and deviation as determined from the fit of the sum spectrums by the AztTecLive Standard software.

	C (wt%)	N (wt%)	Zn (wt%)	Cl (wt%)	O (wt%)	Na (wt%)
ZIF-4-bImCl R=1.0	49.0±0.2	32.8±0.2	10.8±0.0	5.1±0.0	2.3±0.1	0.0±0.0
ZIF-8-bImCl R=1.0	50.2±0.2	28.4±0.2	11.2±0.1	5.0±0.0	3.0±0.1	2.2±0.0
ZIF-62-bImCl R=1.0	51.0±0.2	21.5±0.3	14.8±0.1	7.9±0.0	2.7±0.1	2.2±0.0

Supplementary Table 4

Supplementary Table 4. Peak areas and proton integration ratios from solution-state ^{1}H NMR spectra of digested samples. Proton peaks assigned to benzimidazole (bIm) and imidazole (Im), or to methyl (CH_3) in the case of ZIF-8, were integrated to estimate the relative linker composition in each glass or crystalline sample.

**Sample**	Peak of H 1# (bIm)	Peak of H (H 2#Im or CH_3)	Integration ratio of H from bIm: Im or CH_3 *
ZIF-62-bImCl R=1	1.0	1.16	0.46:0.64 (0:42:0.58)
ZIF-4-bImCl R=1	1.0	1.66	0.38:0.62 (0.33:0.67)
ZIF-62 Crystal	1.0	6.04	0.14:0.86 (0.13:0.87)
ZIF-8-bImCl R=1	1.0	4.37 (CH_3)	0.19:0.81 (0.33:0.67)

*Values in brackets are reference values.

Supplementary Fig. 1

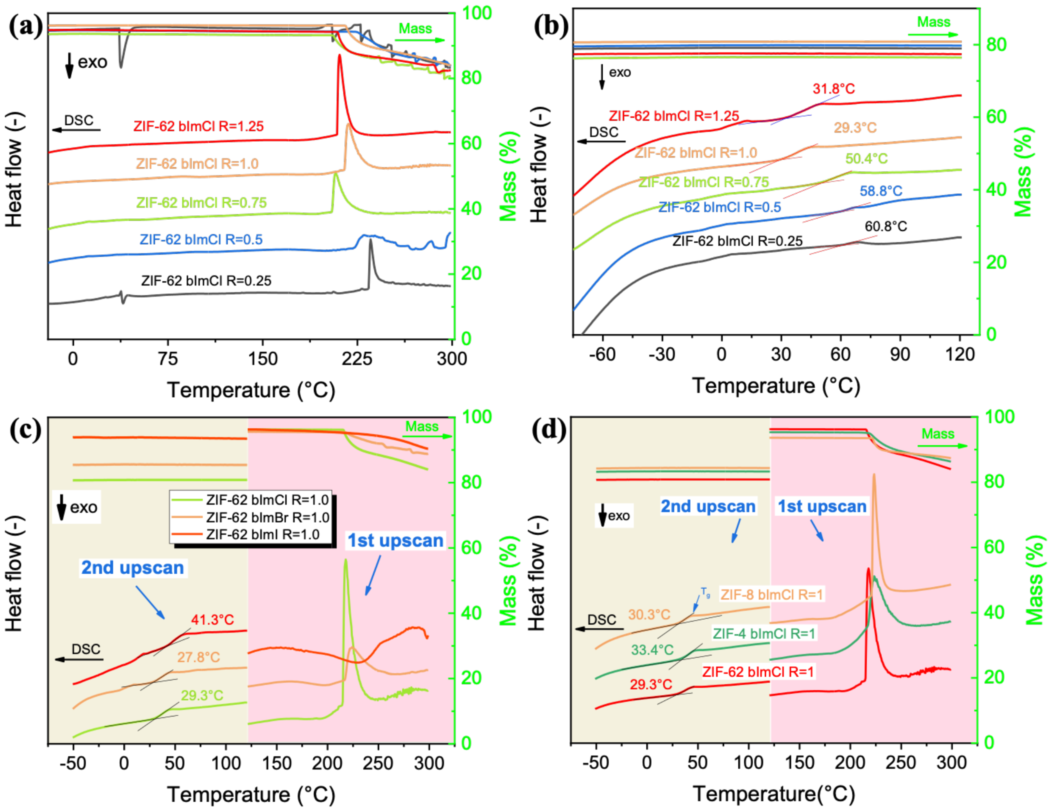

Supplementary Fig. 1. Standard DSC heating upscans at 10 K min^{-1} in Al crucible. (a-b) First (a) and second (b) upscan of ZIF-62 and H_2bImCl with different *R* values (from 0.25 to 1.25). (c) First and second upscan of ZIF-62 with H_2bImCl, H_2bImBr, and H_2bImI at *R* = 1.0. (d) First and second upscan of ZIF-62, ZIF-4, and ZIF-8 with H_2bImCl at *R* = 1.0. Solid lines represent DSC (heat flow) data, while the dash-dotted lines represent the (TGA) mass loss data. Heat flow data have been shifted vertically for clarity. We note that the observed endothermic peaks in panels (a), (c), and (d) correspond to mass loss associated with the bursting of closed crucibles rather than melting, as indicated by the mass loss curves. Notably, the mass remains constant during the second heating scans in panels (b), (c), and (d). In these cases, the endothermic peaks in the DSC curves correspond to the T_g. (Note: A small data jump is observed for the ZIF-62-bImCl R = 0.25 sample in panel (a), which is attributed to an experimental artifact and not a genuine thermal signal from the sample. In addition, a slight mismatch in the mass signal between the end of the first upscan and the beginning of the second upscan is observed due to minor mass loss during the intermediate cooling step.). Source data are provided as a Source Data file.

Supplementary Fig. 2

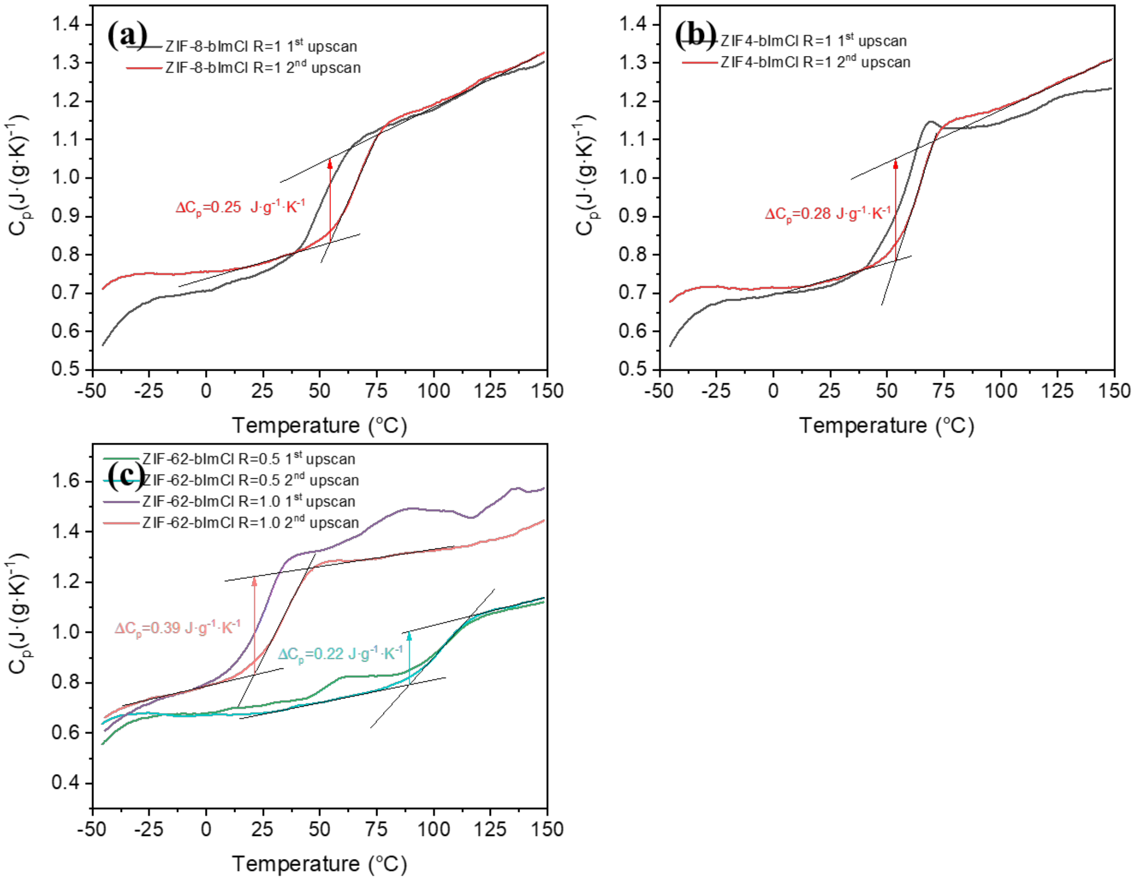

Supplementary Fig. 2. Isobaric heat capacity (C_p) measurements of (a) ZIF-8-bImCl *R*=1.0, (b) ZIF-4-bImCl *R* =1.0, and (c) ZIF-62-bImCl *R*=0.5 and 1.0 samples at a heating and cooling rate of 10 K min^{-1} in PtRh crucibles. The ΔC_p values for ZIF-4-bImCl *R*=1.0 and ZIF-62-bImCl *R*=1.0 are 0.28 and 0.39 $J{\cdot}g^{-1}{\cdot}K^{-1}$, respectively, which are significantly higher than those of unmodified ZIF-4 (0.11 and 0.16 $J{\cdot}g^{-1}{\cdot}K^{-1}$ for the LDA and HDA phases, respectively) and ZIF-62 (0.19 $J{\cdot}g^{-1}{\cdot}K^{-1}$)[4, 5]. Interestingly, ZIF-62-bImCl *R*=0.5 (**c**) exhibits a ΔC_p of 0.22 $J{\cdot}g^{-1}{\cdot}K^{-1}$, which is lower than that of ZIF-62-bImCl *R*=1.0 and close to that of unmodified ZIF-62. These results suggest that increasing the modifier content leads to a higher degree of network depolymerization and configurational entropy, resulting in a more flexible and disordered glassy structure. The relatively lower ΔC_p of ZIF-62-bImCl *R*=0.5 compared to that of *R*=1.0 implies that at lower salt modifier concentrations, the network retains a higher degree of connectivity. Source data are provided as a Source Data file.

Supplementary Fig. 3

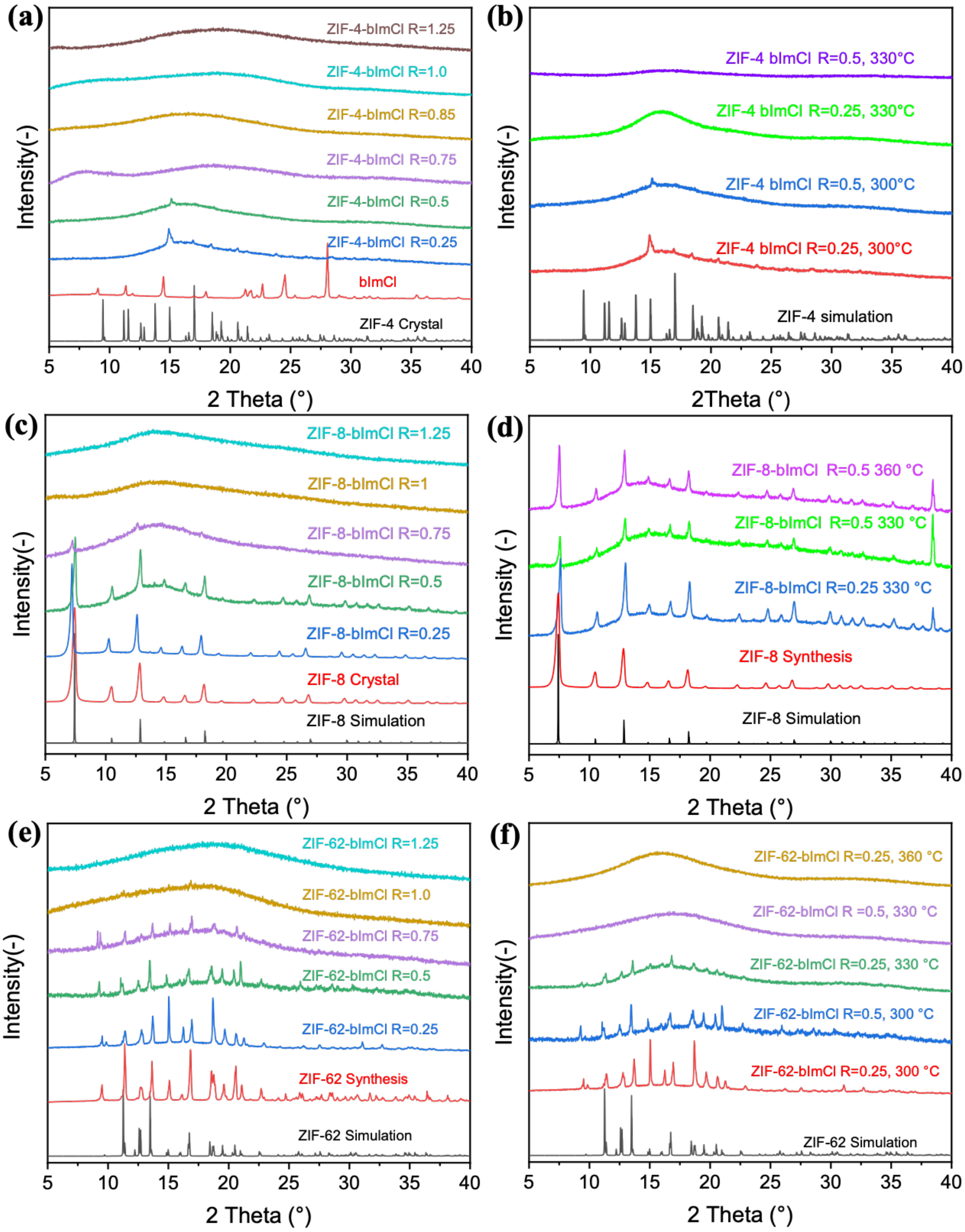

Supplementary Fig. 3. XRD patterns of ZIF-4, ZIF-8, and ZIF-62 with different content of halide salt and heated to varying maximum temperatures: (a) ZIF-4-bImCl (R=0.25~1.25) at T_{max}=300 °C; (b) ZIF-4-bImCl with different molar ratio (R=0.25~0.5) at T_{max}=300-330 °C. (c) ZIF-8-bImCl with different molar ratio (R=0.25~1.25) at T_{max}=300 °C; (d) ZIF-8-bImCl with different molar ratio (R=0.25~0.5) at T_{max}=300-360 °C. (e) ZIF-62-bImCl with R=0.25~1.25) ratio at T_{max}=300 °C; (f) ZIF-62-bImCl with R=0.25~0.5 ratio at T_{max}=300-360 °C. Source data are provided as a Source Data file.

Supplementary Fig. 4

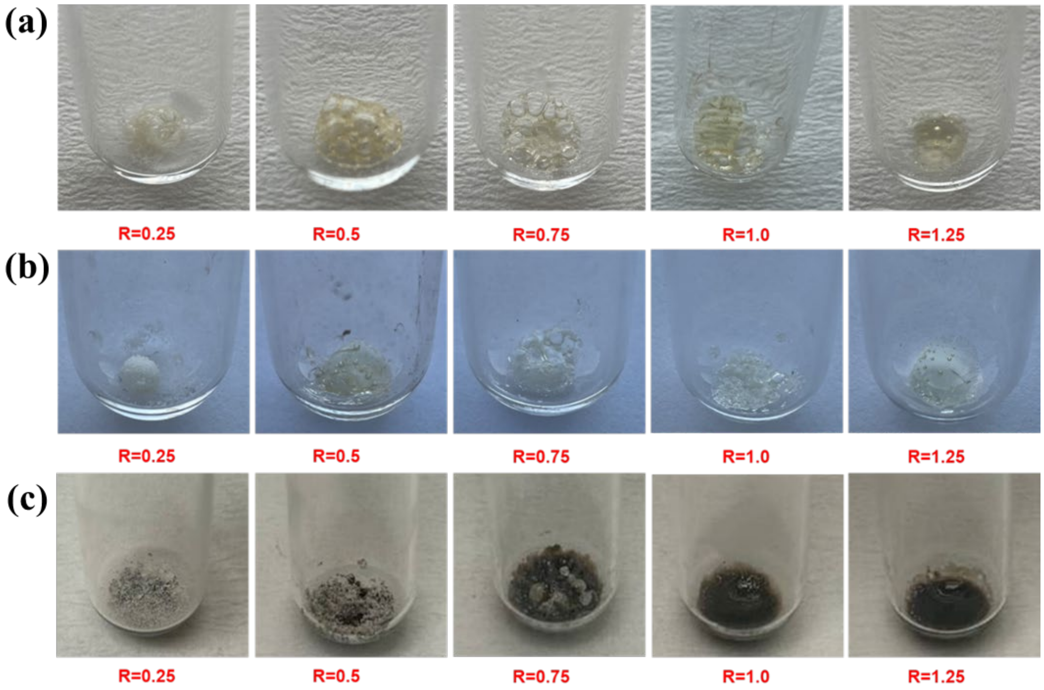

Supplementary Fig. 4. Images of the recovered ZIF-halide salt samples after melt-quenching: (a) ZIF-4-bImCl, (b) ZIF-62-bImCl, and (c) ZIF-8-bImCl with different molar proportions of H_2bImCl (*R*). All samples were heated to a maximum temperature of 300 °C.

Supplementary Fig. 5

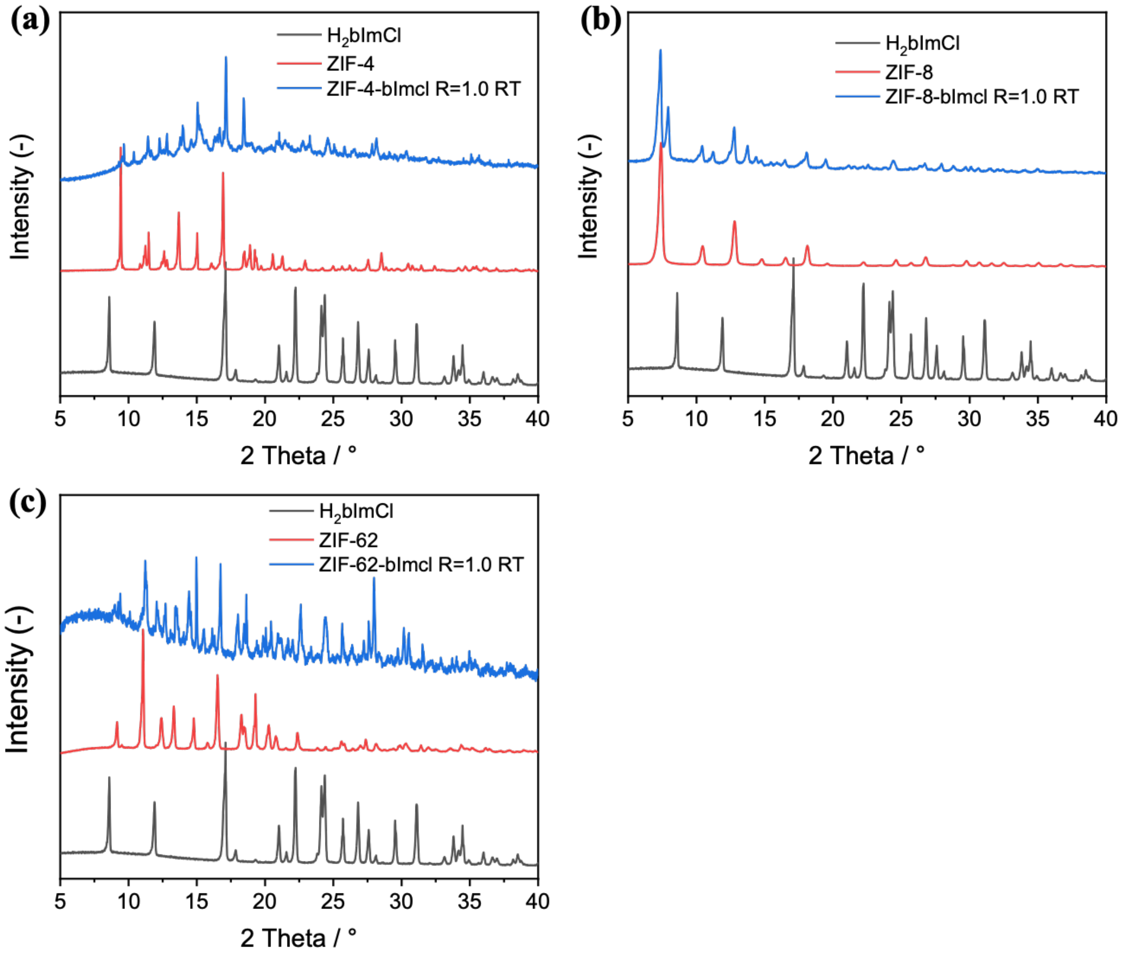

Supplementary Fig. 5. XRD patterns of the mixture of salt (H_2bImCl) and crystal at room temperature (RT): (a) ZIF-4-bImCl *R*=1.0, (b) ZIF-8-bImCl *R*=1.0, and (c) ZIF-62-bImCl *R*=1.0. Source data are provided as a Source Data file.

Supplementary Fig. 6

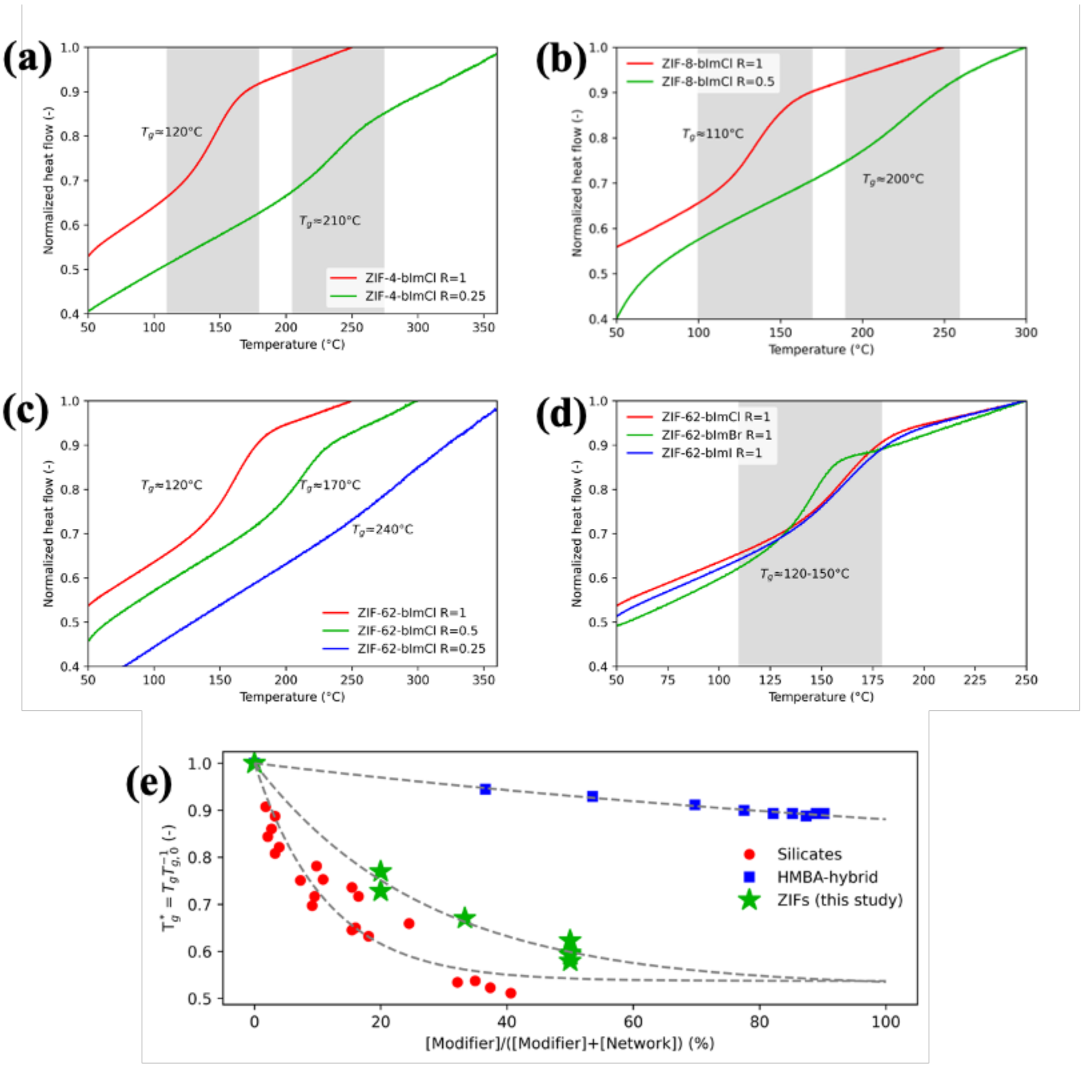

Supplementary Fig. 6. (a-d) Fast scanning calorimetry data of (a) ZIF-4-bImCl, (b) ZIF-8-bImCl, (c) ZIF-62bImCl, and (d) ZIF-62-bIm[Cl, Br, I] samples. All scans were performed at 500 K s^{-1}. The difference in the width of the glass transition may be ascribed to a combination of sample size differences as well as differences in fragility whereas higher fragility may produce broader glass transitions. (e) Effect of modifier content on the reduced glass transition temperature ($T_g^* = T_g T_{g,0}^{-1}$, where T_g is the glass transition of a modified glass and $T_{g,0}$ is the glass transition temperature of a pure glass former) for the present ZIFs as well as literature data for silicates and other hybrid glasses (HMBA: *N*,*N'*-1,6-hexamethylenebis-acetamide)[6]. Note that the normalization is different compared to Figure 1d in the main text. The dashed line indicates a guide for the eye as obtained from fits to exponential decay functions. Source data are provided as a Source Data file.

Supplementary Fig. 7

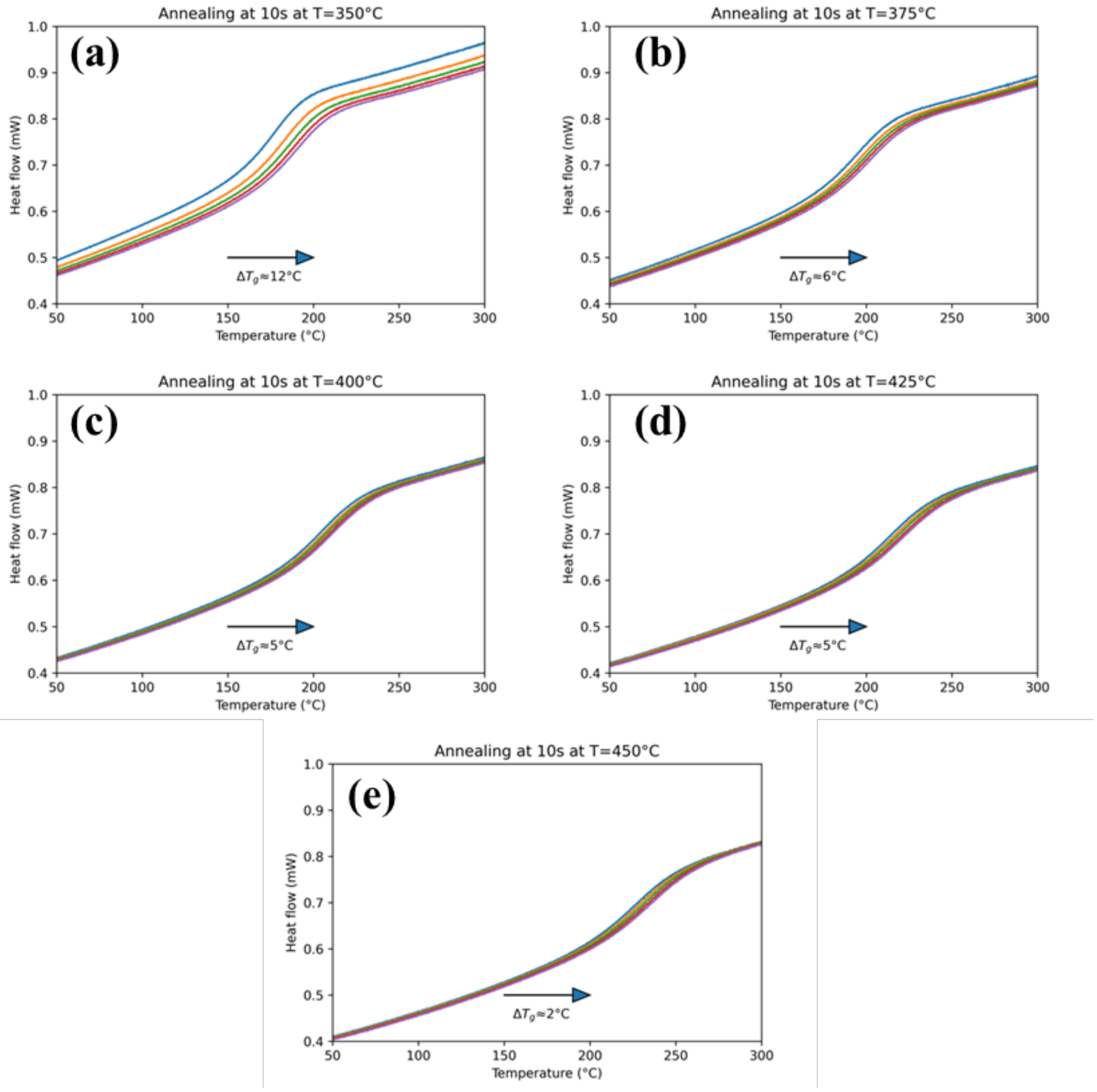

Supplementary Fig. 7. FDSC annealing test of a ZIF-62-bImCl (*R*=1.0) sample subjected to five consecutive annealing treatments, each of 10 s, at temperatures of (a) 350, (b) 375, (c) 400, (d) 425, and (e) 450 °C. The annealing treatments were performed in the given order on the same glass sample. A total increase in T_g of ~30 °C is observed. All scans were performed at rates of 500 K s^{-1}. Source data are provided as a Source Data file.

Supplementary Fig. 8

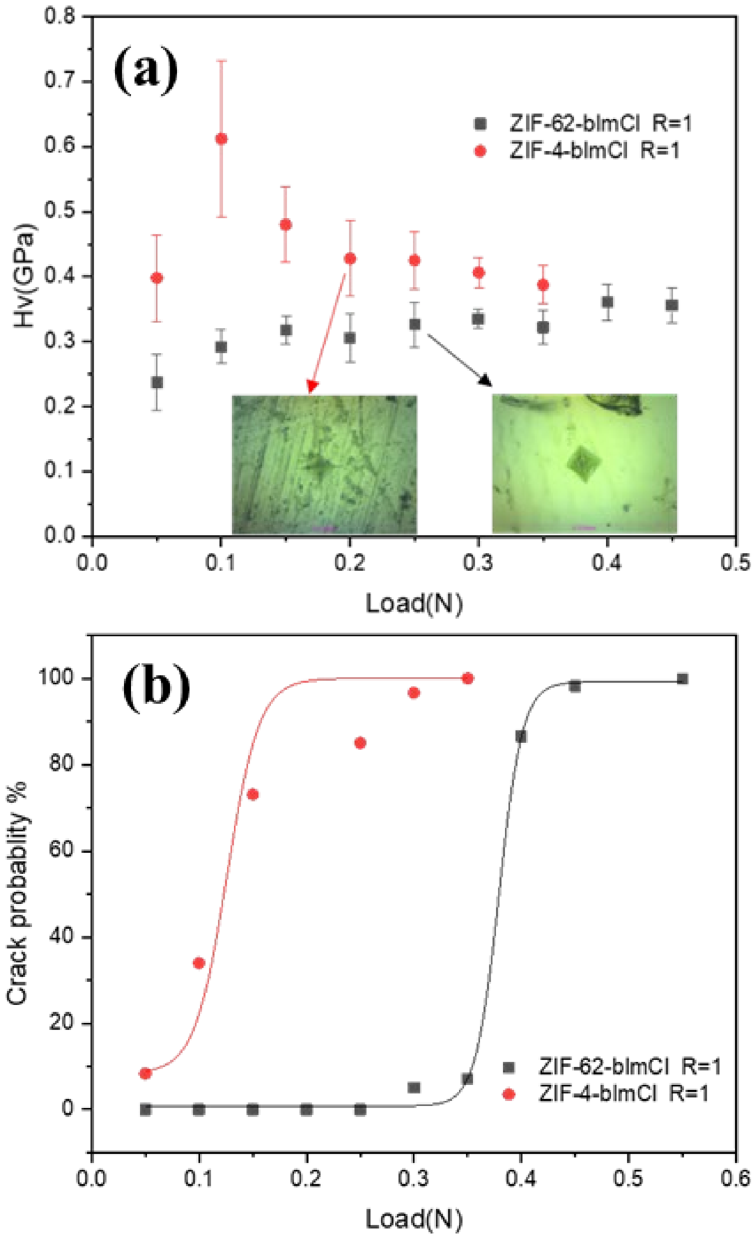

Supplementary Fig. 8. (a) Vickers hardness (H_V) of ZIF-4-bImCl and ZIF-62-bImCl R=1 glasses (error bars represent standard deviations). Insets: examples of indent morphologies. (b) Crack initiation probability as a function of applied Vickers indentation load for the same glasses as in panel (a). The lines represent sigmodal fits to the data. Source data are provided as a Source Data file.

Supplementary Fig. 9

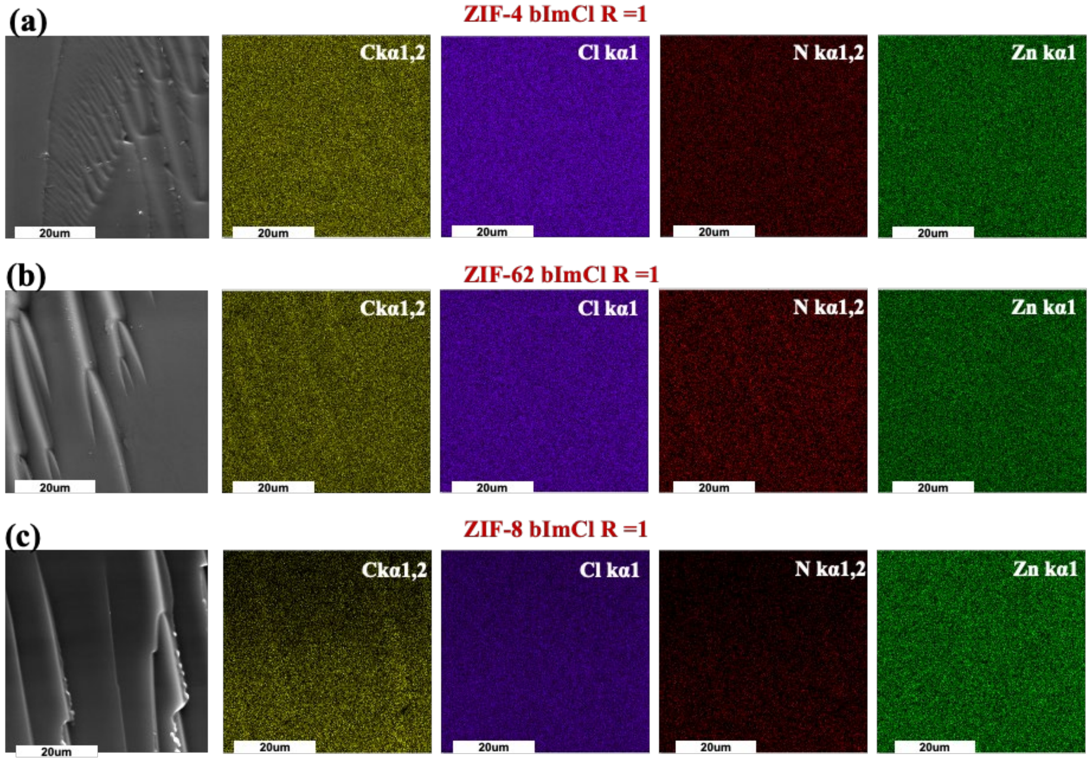

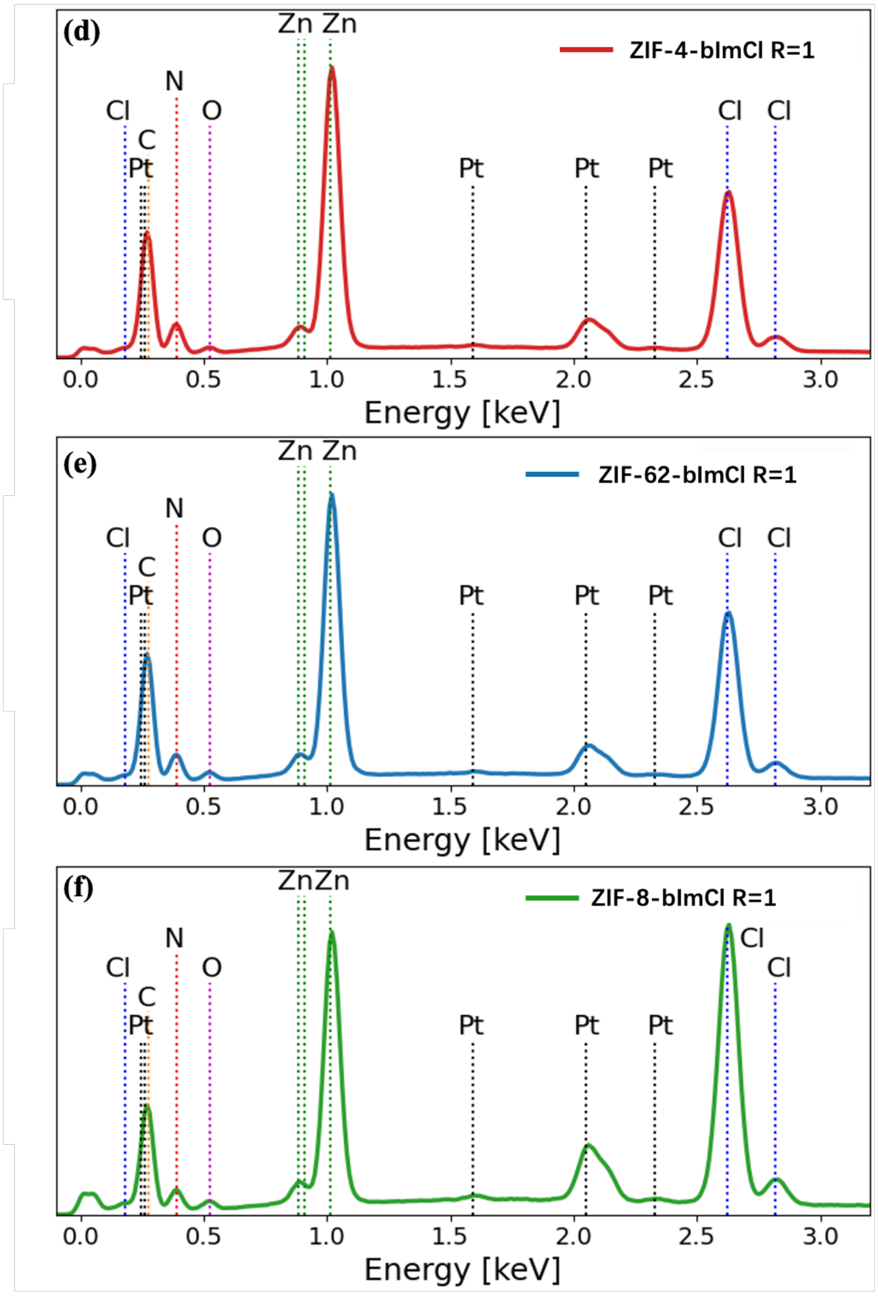

Supplementary Fig. 9. (a-c) Scanning electron microscopy (SEM) images and energy dispersive X-ray (EDX) compositional maps for (a) ZIF-4-bImCl, (b) ZIF-62-bImCl, and (c) ZIF-8-bImCl glasses (all *R*=1.0). (d-f) EDX sum spectra for (d) ZIF-4-bImCl (e) ZIF-62-bImCl, and (f) ZIF-8-bImCl glasses (all *R*=1.0). Characteristic peaks corresponding to C, N, O, Cl, Zn, and Pt (from conductive coating) are indicated. Source data are provided as a Source Data file.

Supplementary Fig. 10

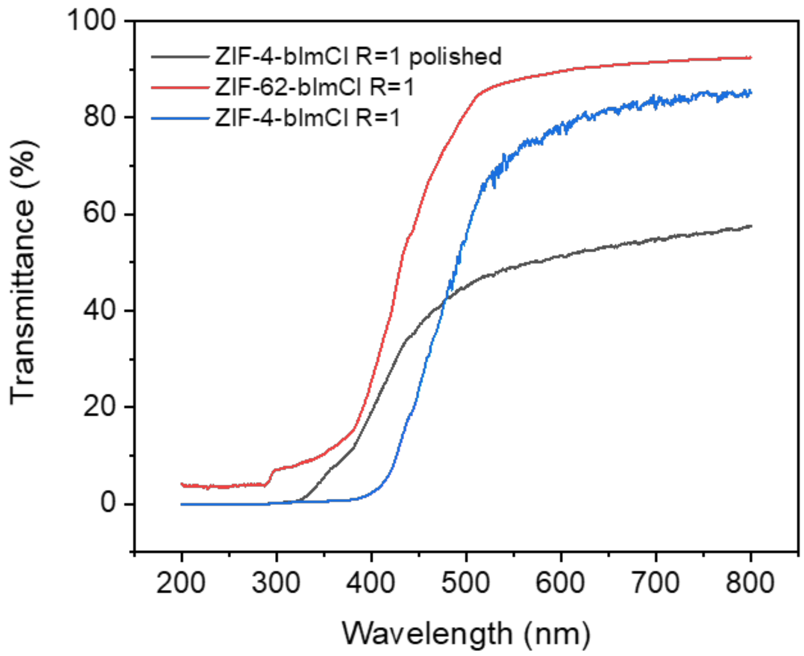

Supplementary Fig. 10. Optical transmission data of ZIF-4-bImCl and ZIF-62-bImCl *R*=1 glasses (normalized to a thickness of 1.0 mm). The difference between polished and as-prepared ZIF-4-bImCl glass is also highlighted, showing the difficulty of preparing a proper polished surface of these very soft materials. Source data are provided as a Source Data file.

Supplementary Fig. 11

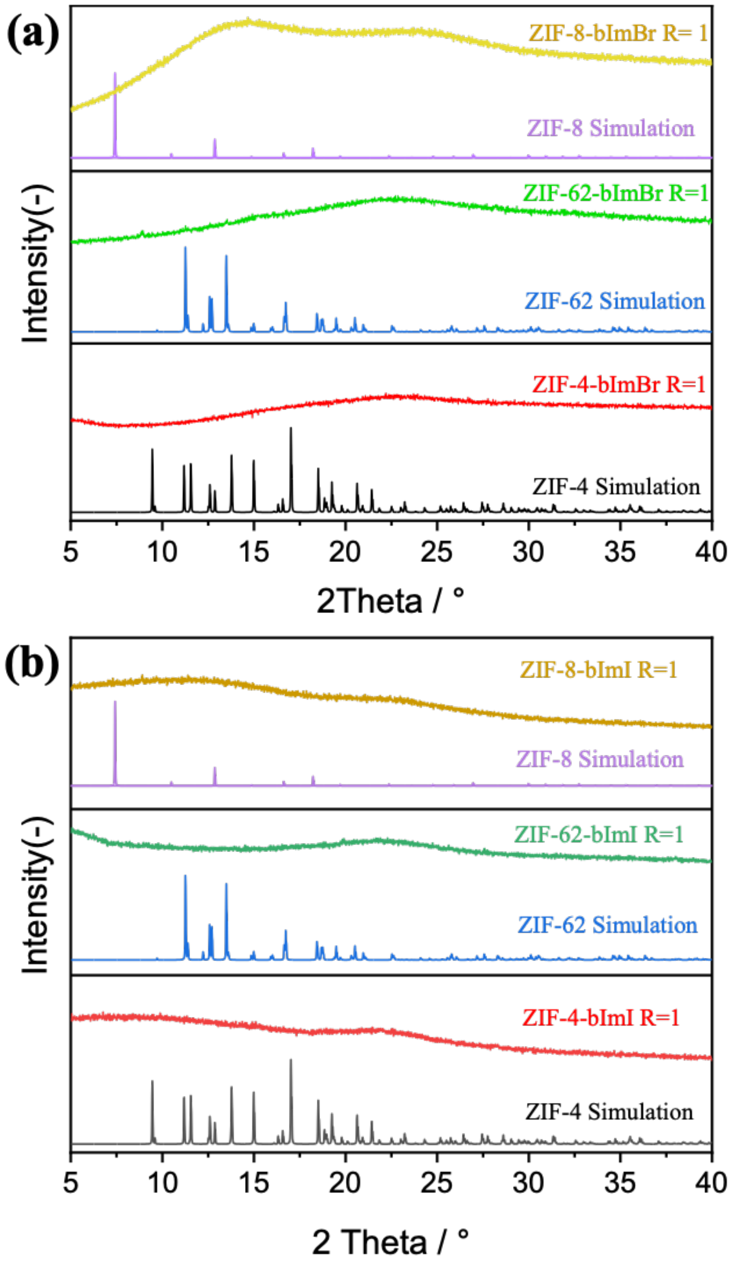

Supplementary Fig. 11. (a) X-ray diffraction (XRD) patterns of bImBr modified ZIF-4, ZIF-62, and ZIF-8 glasses (all *R*=1.0). (b) XRD patterns of bImI modified ZIF-4, ZIF-62, and ZIF-8 glasses (all *R*=1.0). Source data are provided as a Source Data file.

Supplementary Fig. 12

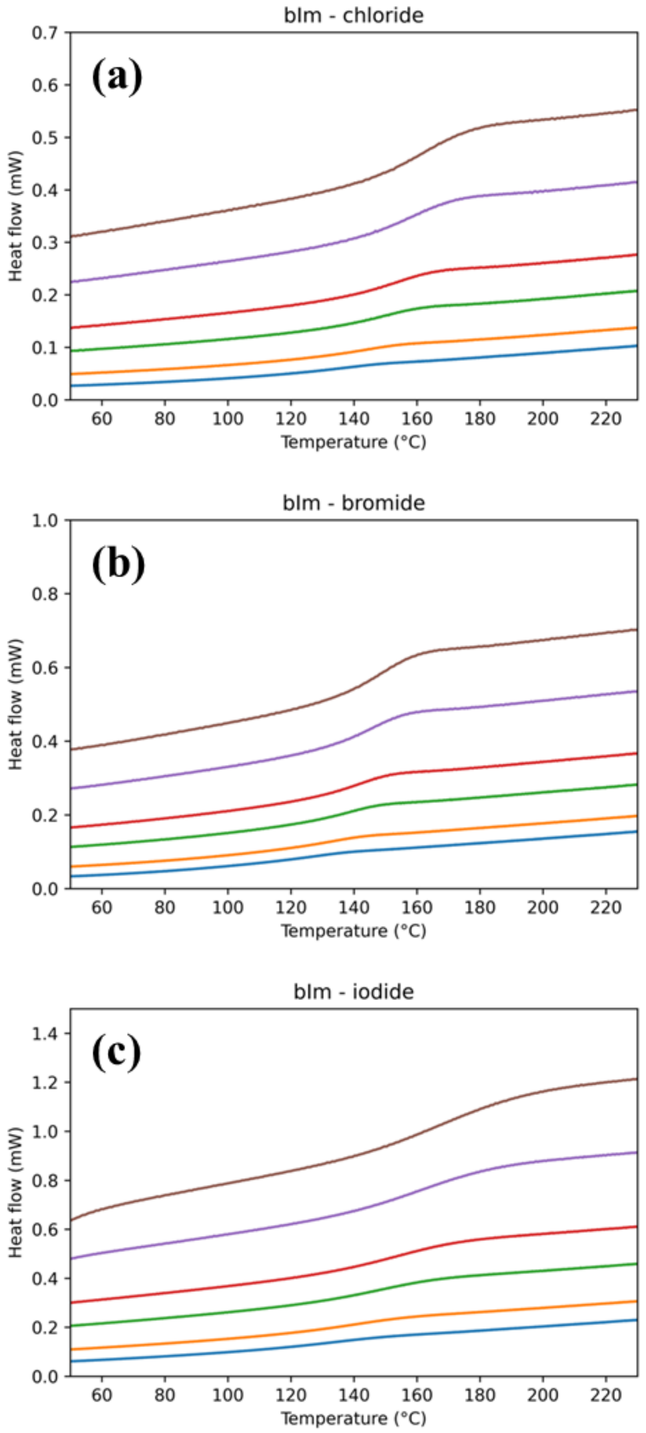

Supplementary Fig. 12. Fast scanning calorimetry data of (a) ZIF-62-bImCl, (b) ZIF-62-bImBr, and (c) ZIF-62-bImI samples (all R=1). Sample scans are performed at 50 (blue lines), 100 (yellow), 200 (green), 300 (red), 500 (purple), and 700 (brown) K s^{-1}, respectively. Higher heating rates give rise to higher heat flow values for all samples. Source data are provided as a Source Data file.

Supplementary Fig. 13

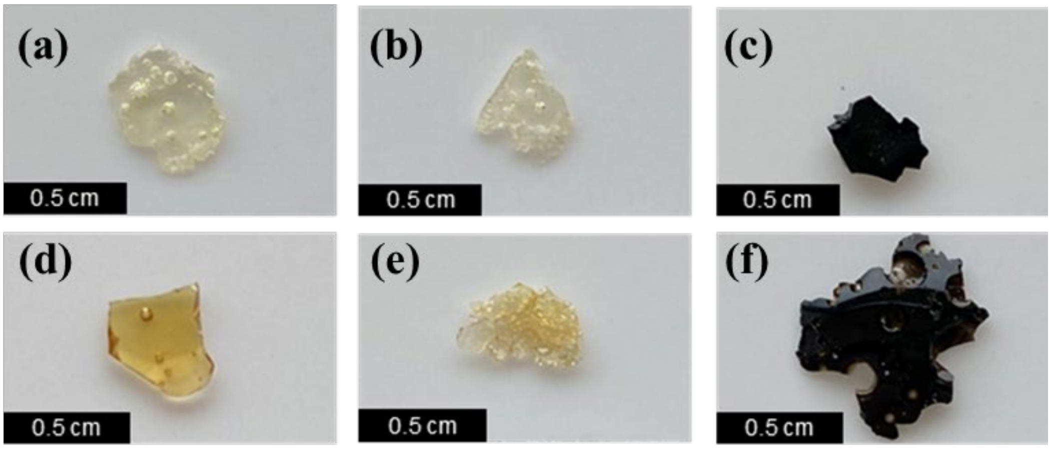

Supplementary Fig. 13. (a-c) Images of bImBr modified (a) ZIF-4, (b) ZIF-62, and (c) ZIF-8 glasses (*R*=1). (d-f) Images of bImI modified (d) ZIF-4, (e) ZIF-62, and (f) ZIF-8 glasses (*R*=1). All samples were prepared at 300 ºC. Source data are provided as a Source Data file.

Supplementary Fig. 14

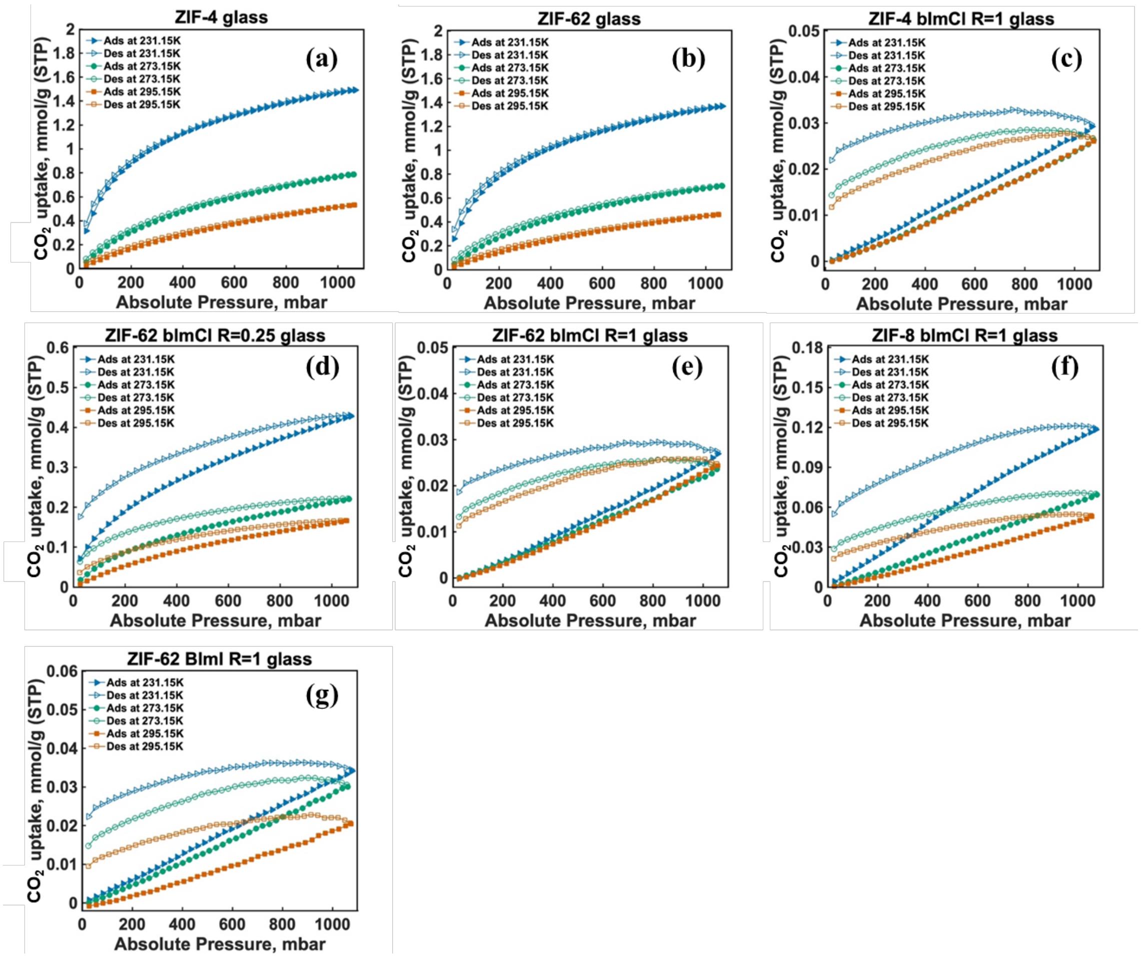

Supplementary Fig. 14. CO_2 adsorption isotherms for selected samples at temperatures of 231 K (-42 °C), 273 K (0 °C), and 295 K (22 °C, RT): (a) ZIF-4 glass, (b) ZIF-62 glass, (c) ZIF-4-bImCl *R*=1 glass, (d) ZIF-62-bImCl *R*=0.25 glass, (e) ZIF-62-bImCl *R*=1 glass, (f) ZIF-8-bImCl *R*=1 glass, and (g) ZIF-62-bImI *R*=1 glass. We note that the *y*-axis range of the plots vary among the different samples. Source data are provided as a Source Data file.

Supplementary Fig. 15

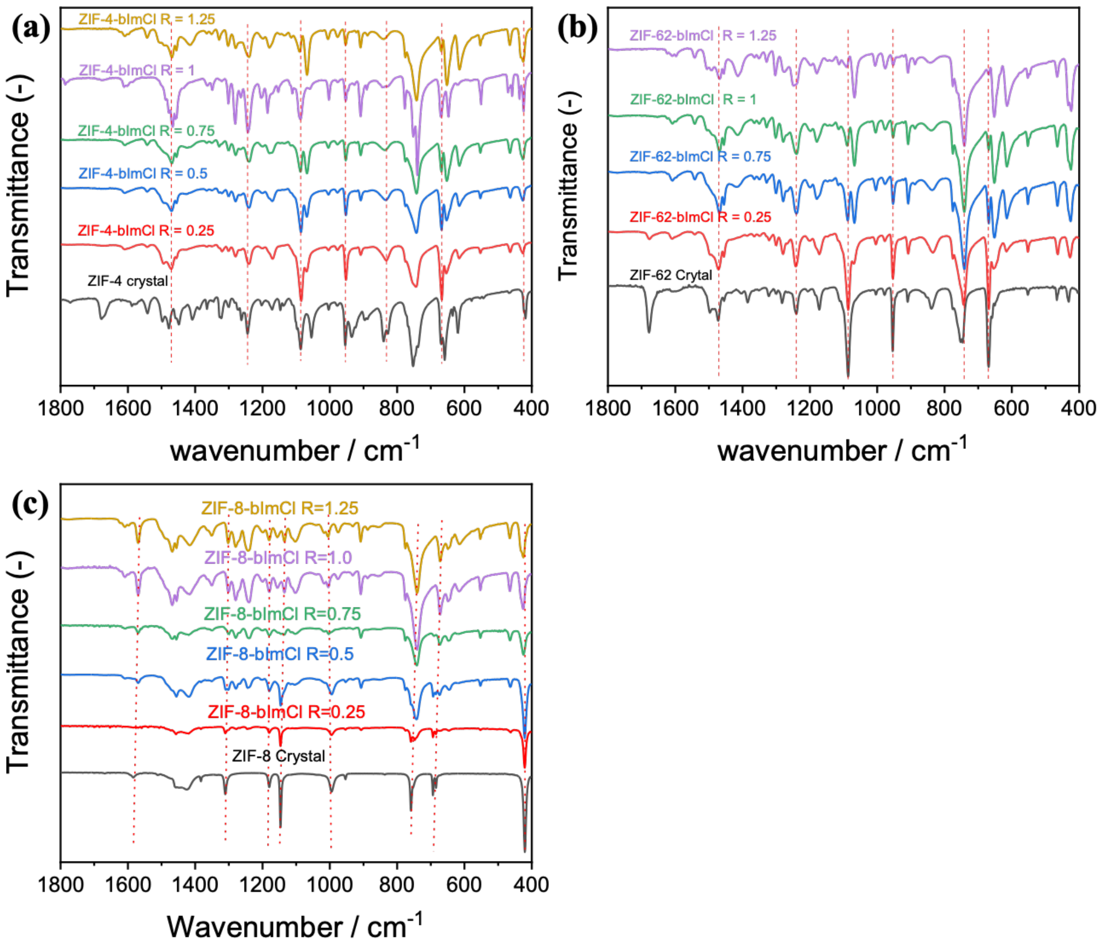

Supplementary Fig. 15. FT-IR spectroscopy transmission data of the modified samples with different *R* values: (a) ZIF-4-bImCl (*R*= 0.25~1.25); (b) ZIF-62-bImCl (*R*= 0.25~1.25); (c) ZIF-8-bImCl (*R*= 0.25~1.25). Source data are provided as a Source Data file.

Supplementary Fig. 16

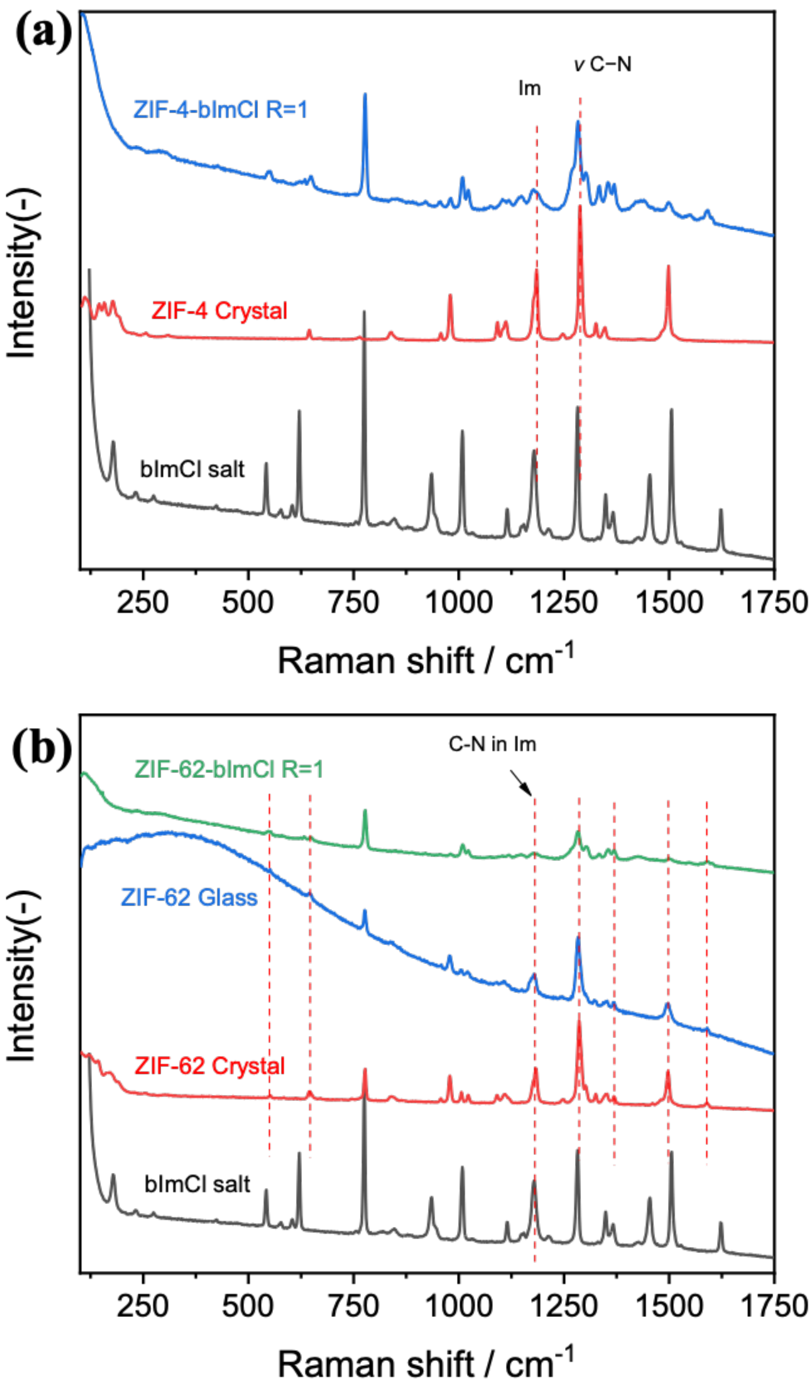

Supplementary Fig. 16. Raman spectroscopy data of (a) crystalline ZIF-4 and glassy ZIF-4-bImCl R=1 as well as (b) crystalline ZIF-62 and glassy ZIF-62-bImCl R=1. In both panels (a) and (b), the spectrum of the pure H_2bImCl salt is shown. Source data are provided as a Source Data file.

Supplementary Fig. 17

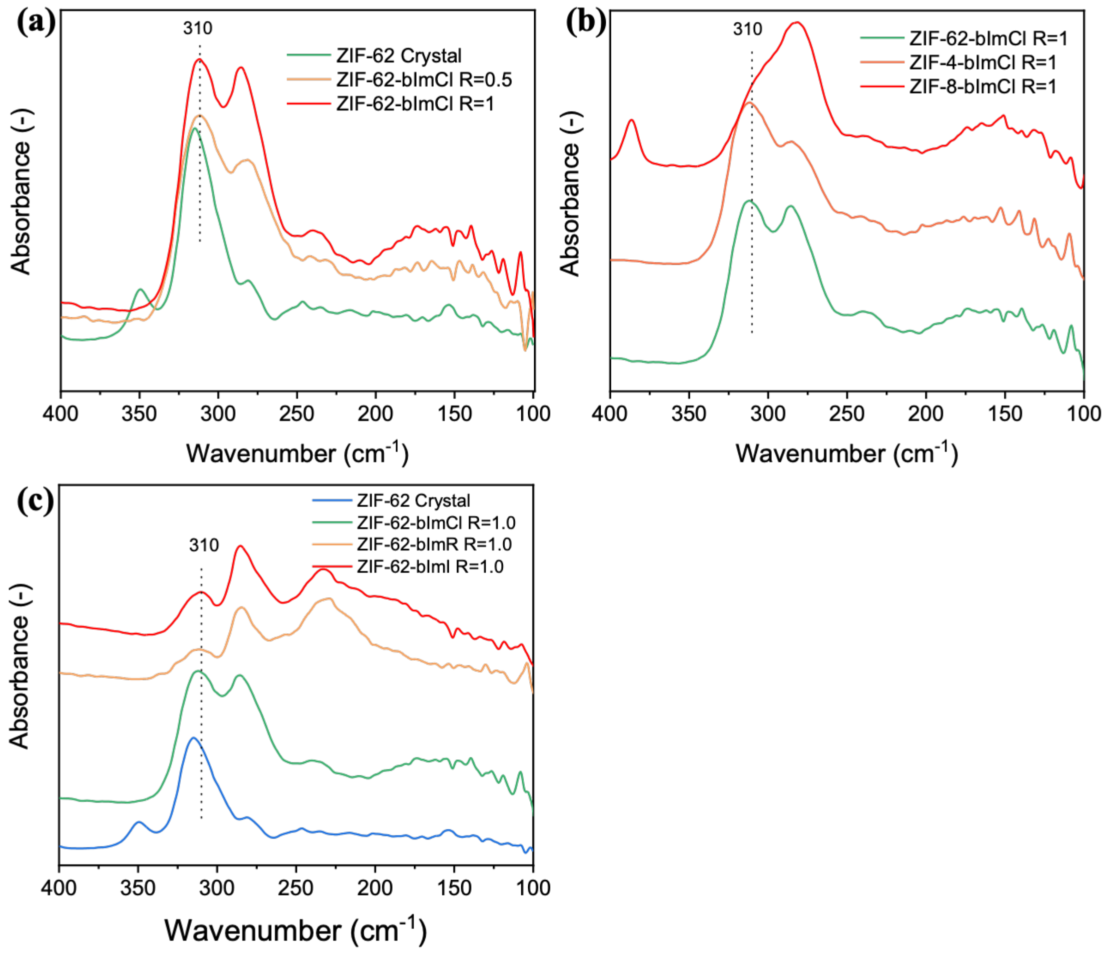

Supplementary Fig. 17. Far-infrared (FIR) spectroscopy measurements of (a) ZIF-62-bImCl glasses with different *R* values (0.5 and 1.0), (b) ZIF-62/4/8-bImCl glasses with constant *R*=1.0, and (c) ZIF-62-bImCl/Br/I glasses with constant *R*=1.0. Source data are provided as a Source Data file.

Supplementary Fig. 18

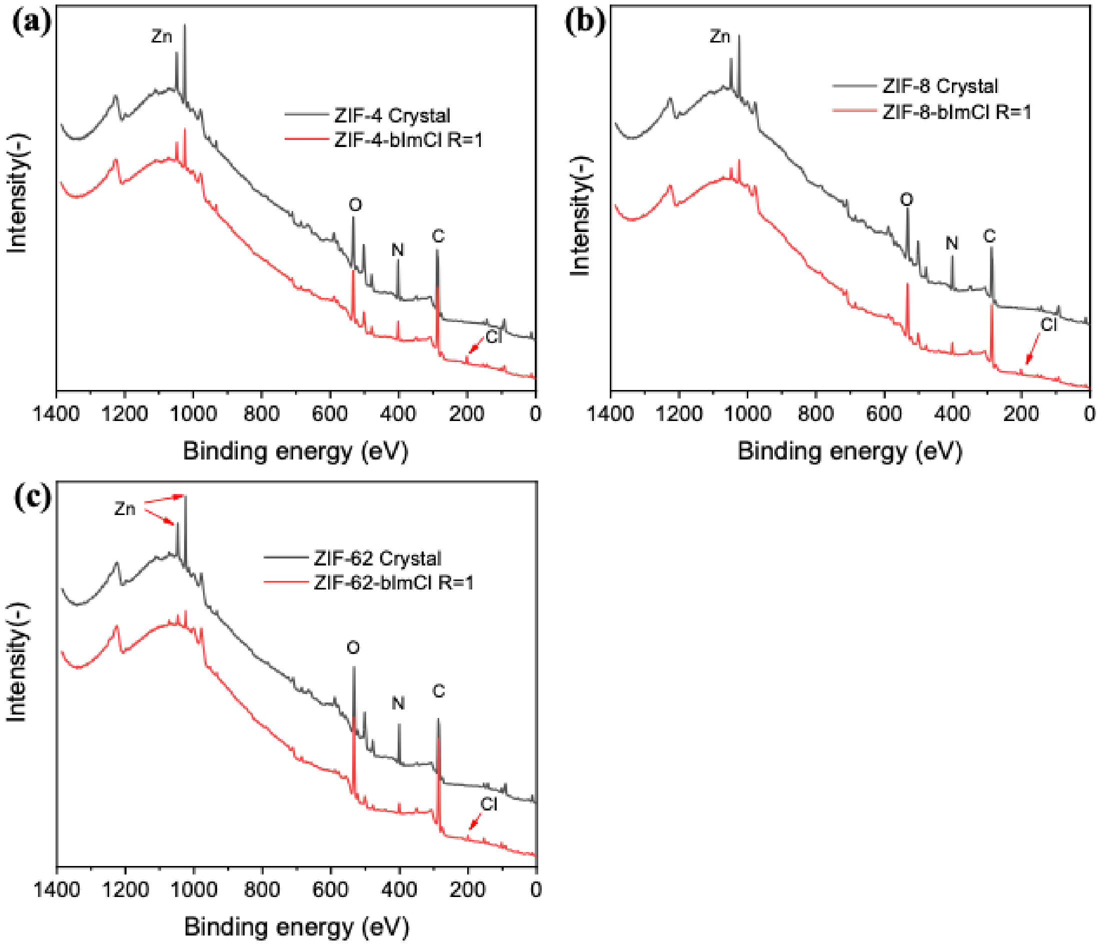

Supplementary Fig. 18. XPS spectra of the ZIF crystals as well as ZIF-bImCl glass. (a) ZIF-4 crystal and ZIF-4-bImCl *R*=1 glass; (b) ZIF-8 crystal and ZIF-8-bImCl *R*=1 glass; (c) ZIF-62 crystal and ZIF-62-bImCl *R*=1 glass. Source data are provided as a Source Data file.

Supplementary Fig. 19

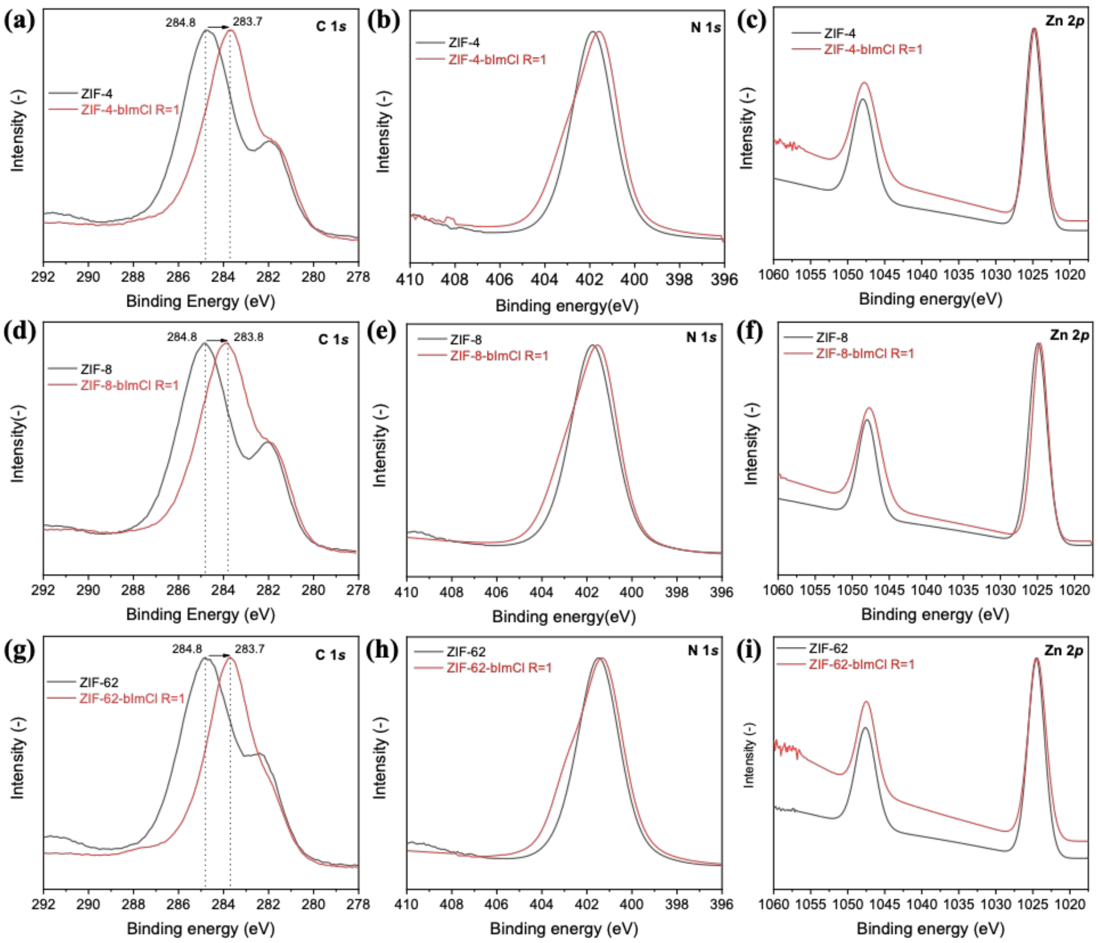

Supplementary Fig. 19. (a-c) XPS data for (a) C 1*s*, (b) N 1*s*, and (c) Zn 2*p* of ZIF-4 and ZIF-4-bImCl *R*=1 glasses. (d-f) XPS data for (d) C 1*s*, (e) N 1*s*, and (f) Zn 2*p* of ZIF-8 and ZIF-8-bImCl *R*=1 glasses. (g-i) XPS data for (g) C 1*s*, (h) N 1*s*, and (i) Zn 2*p* of ZIF-62 and ZIF-62-bImCl *R*=1 glasses. Source data are provided as a Source Data file.

Supplementary Fig. 20

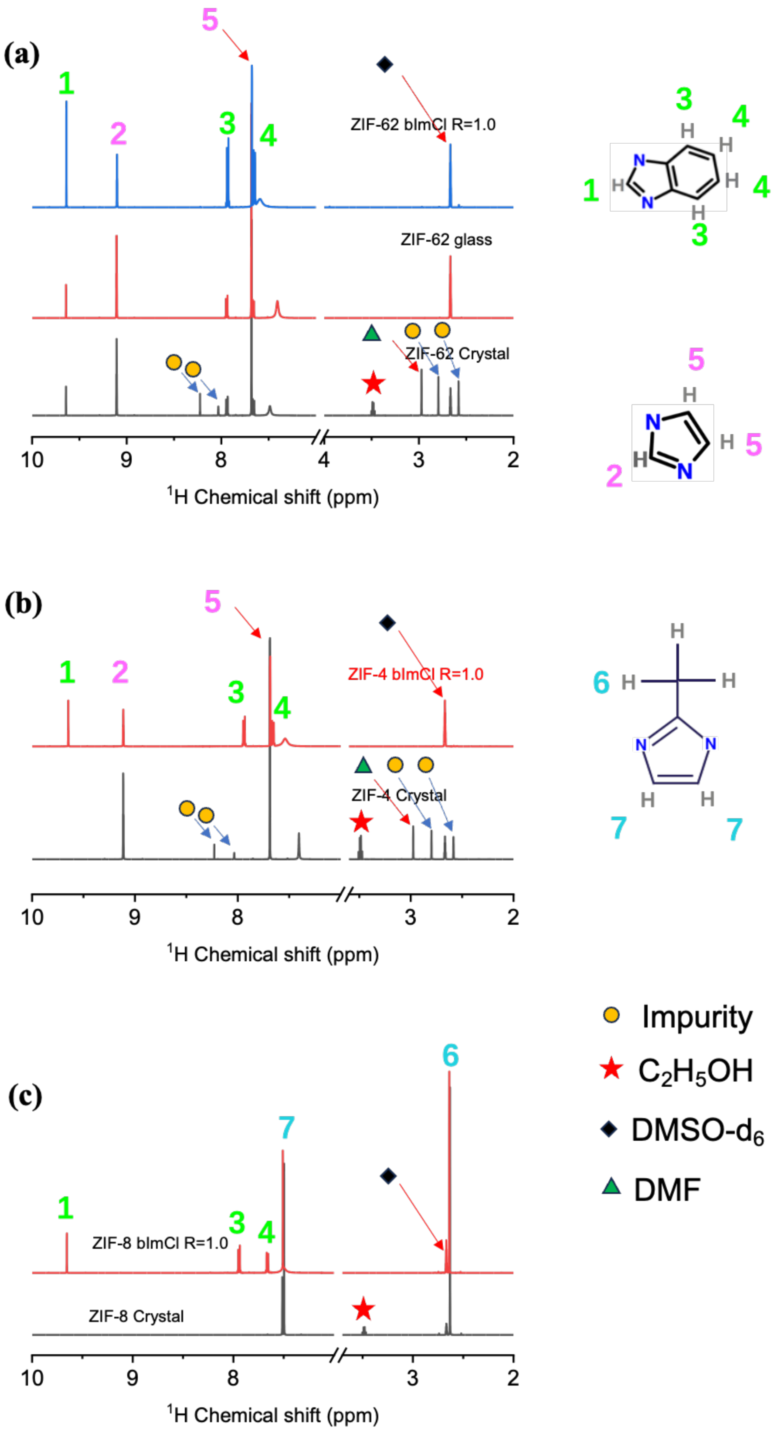

Supplementary Fig. 20. Solution ^{1}H NMR spectra of (a) ZIF-62 crystal, ZIF-62 glass, and ZIF-62-bImCl *R*=1, (b) ZIF-4 crystal and ZIF-4-bImCl *R*=1, and (c) ZIF-8 crystal and ZIF-8-bImCl *R*=1 samples. Integration ratios are given in Supplementary Table 4. Source data are provided as a Source Data file.

Supplementary Fig. 21

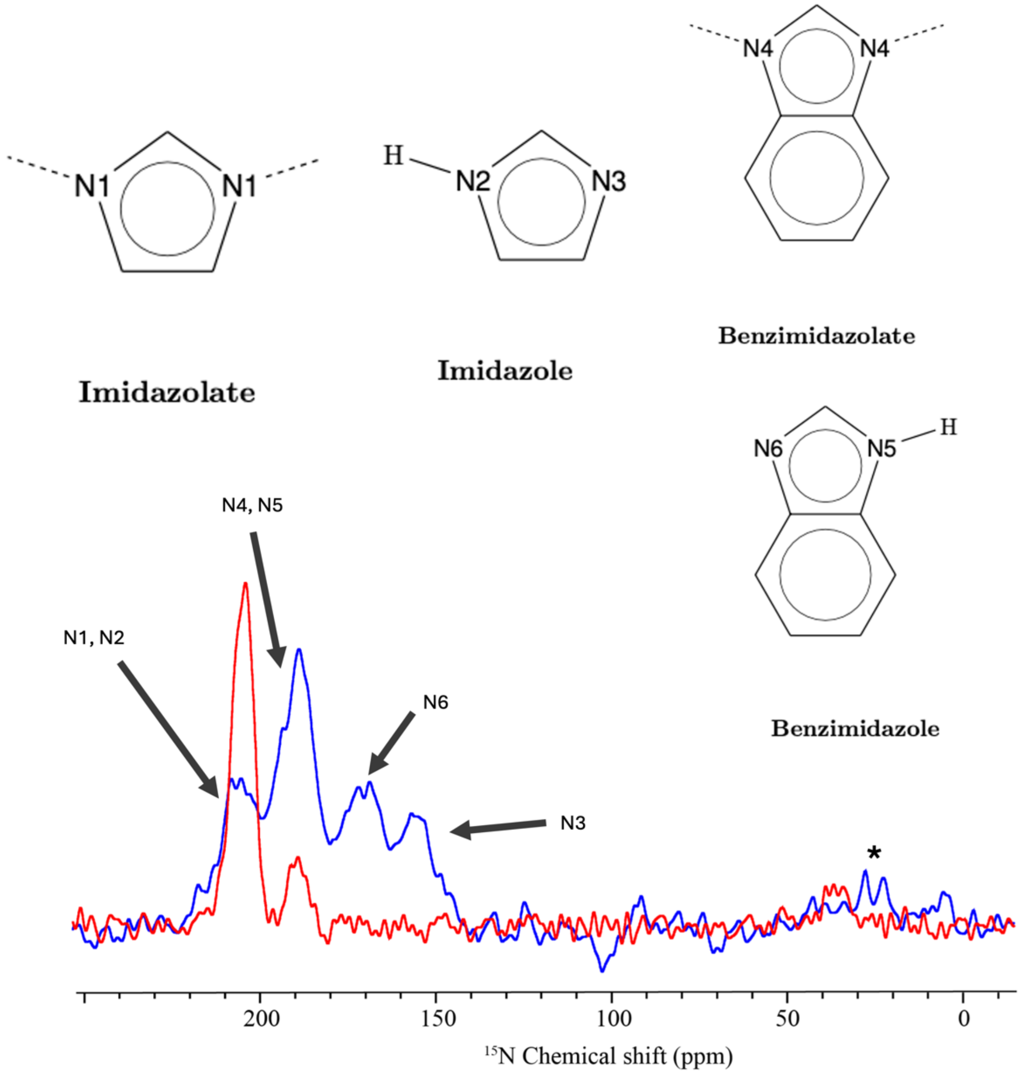

Supplementary Fig. 21. Solid-state ^{15}N MAS NMR spectra of (red) ZIF-62 and (blue) ZIF-62-bImCl *R*=1 glasses. The chemical shifts of imidazolate, benzimidazolate, and their protonated versions are interpreted based on previous measurements in Supporting Refs.[7, 8, 9, 10] A spinning sideband is denoted with '*'. Source data are provided as a Source Data file.

Supplementary Fig. 22

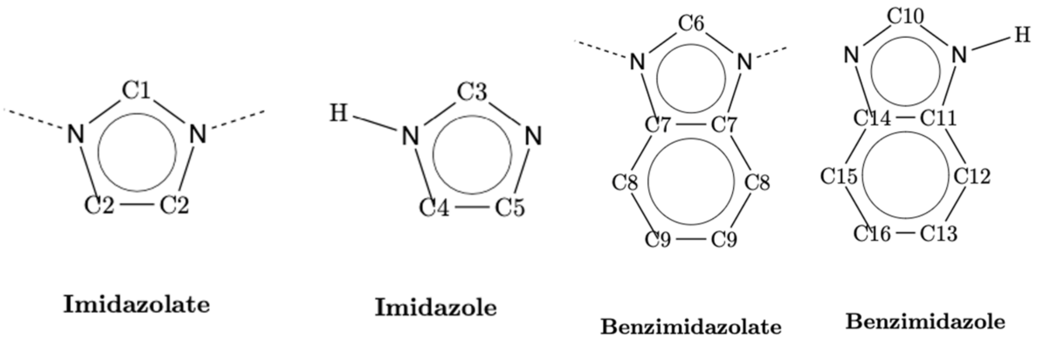

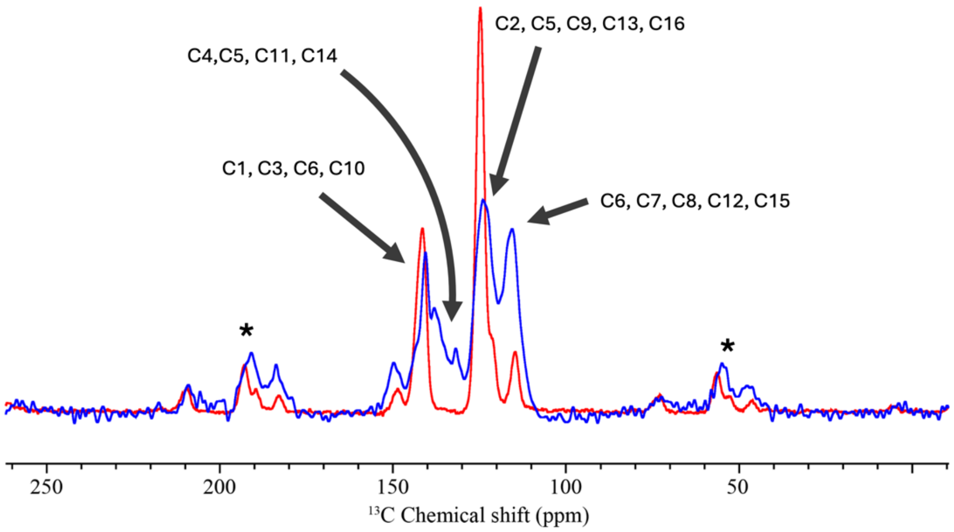

Supplementary Fig. 22. Solid-state ^{13}C MAS NMR spectra for (red) ZIF-62 and (blue) ZIF-62-bImCl *R*=1 glasses. The chemical shifts of imidazolate, benzimidazolate, and their protonated versions are interpreted based on previous measurements in Supporting Ref.[7] Spinning sidebands are denoted with '*'. Source data are provided as a Source Data file.

Supplementary Fig. 23

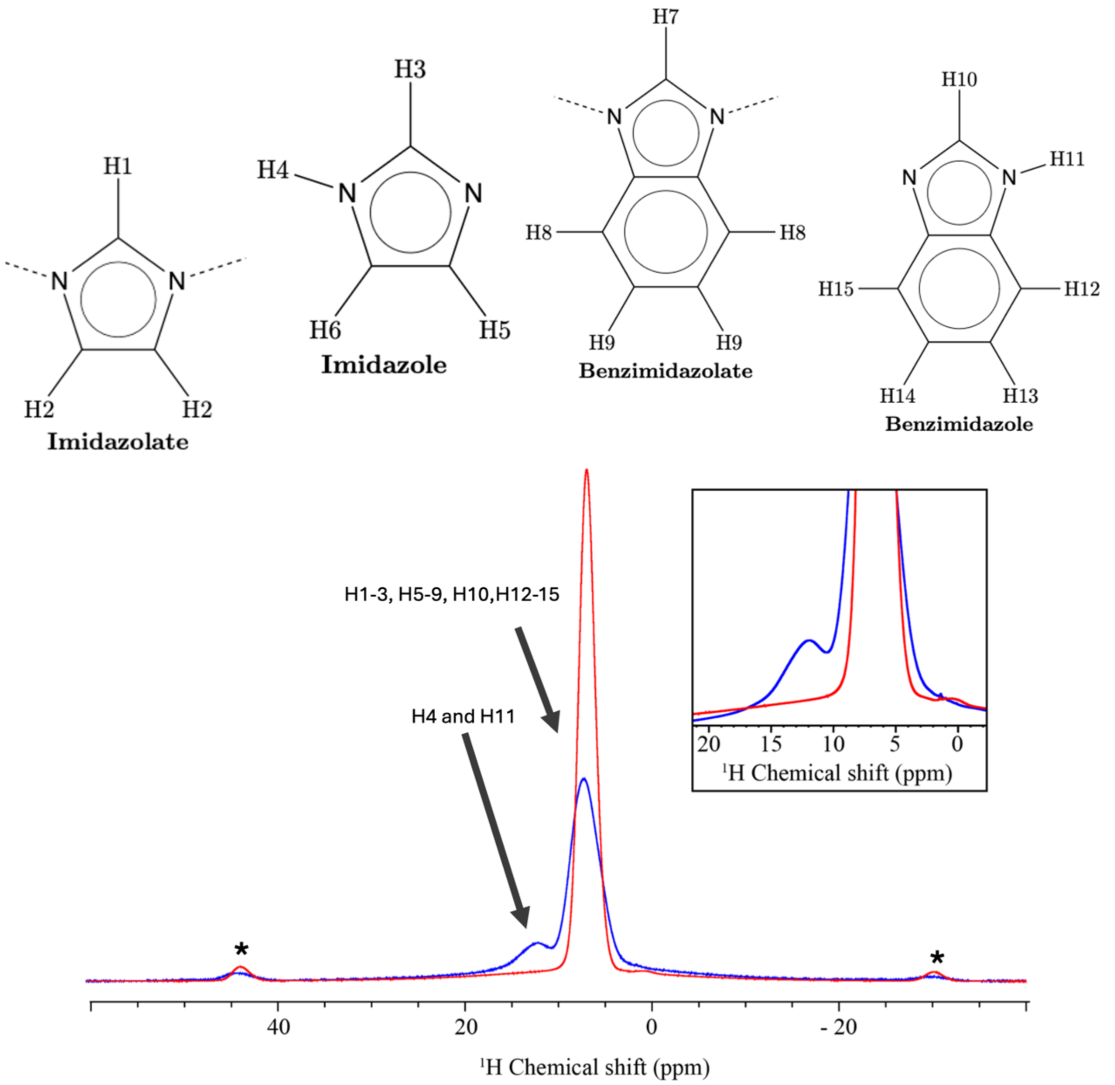

Supplementary Fig. 23. Solid-state ^{1}H MAS NMR spectra of (red) ZIF-62 and (blue) ZIF-62-bImCl *R*=1 glasses. The chemical shifts of the proton in imidazole/benzimidazole are based on previous measurements of Supporting Ref.[7] Spinning sidebands are denoted with '*'. Source data are provided as a Source Data file.

Supplementary Fig. 24

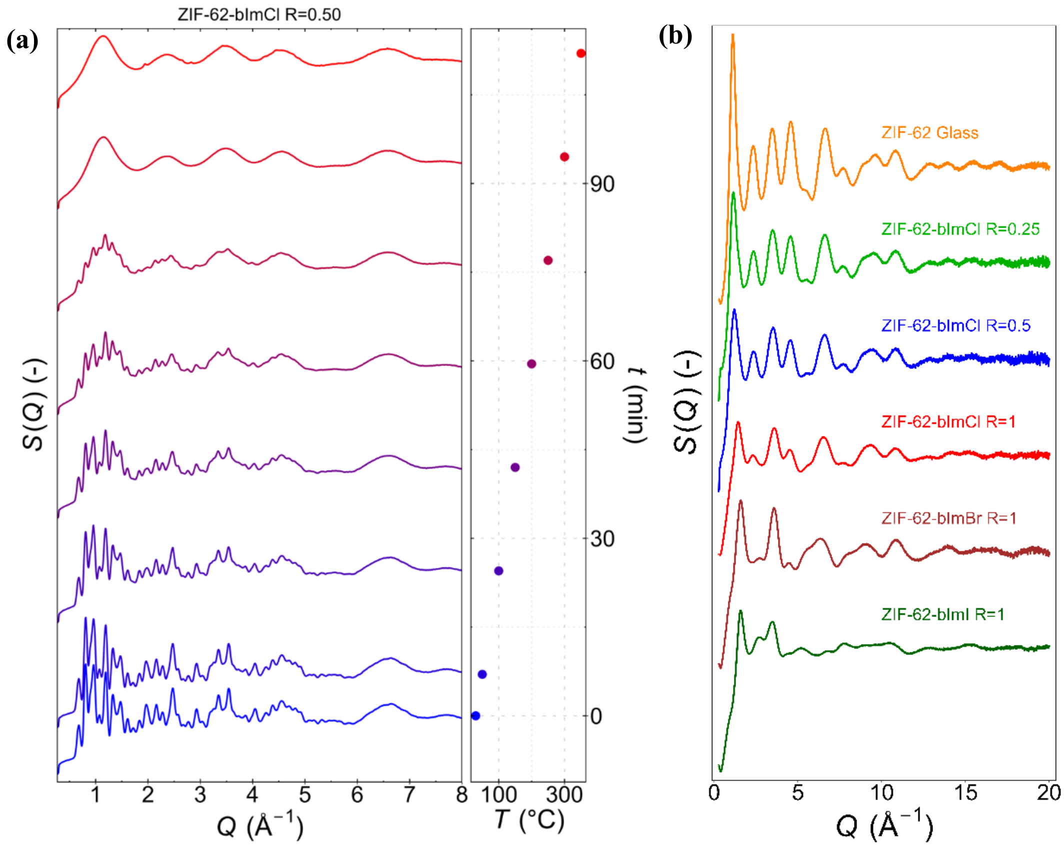

Supplementary Fig. 24. (a) *In situ* X-ray total scanning variable temperature structure factor $S(Q)$ data for ZIF-62-bImCl R=0.5. The measurements start from the initially mixed crystals (t=0). The loss of Bragg peaks is noticed at around 300 °C. Data presented here corresponds to the same temperature points as presented in Figure 3b in the main text. Further data points for both $S(Q)$ and $G(r)$ are presented in Supplementary Fig. 26. (b) X-ray total structure factor $S(Q)$ data for the glasses; ZIF-62, ZIF-62-bImCl (R=0.25~1.0), ZIF-62-bImBr R=1, and ZIF-62-bImI R=1. All measurements in panel (b) were performed at room temperature. Source data are provided as a Source Data file.

Supplementary Fig. 25

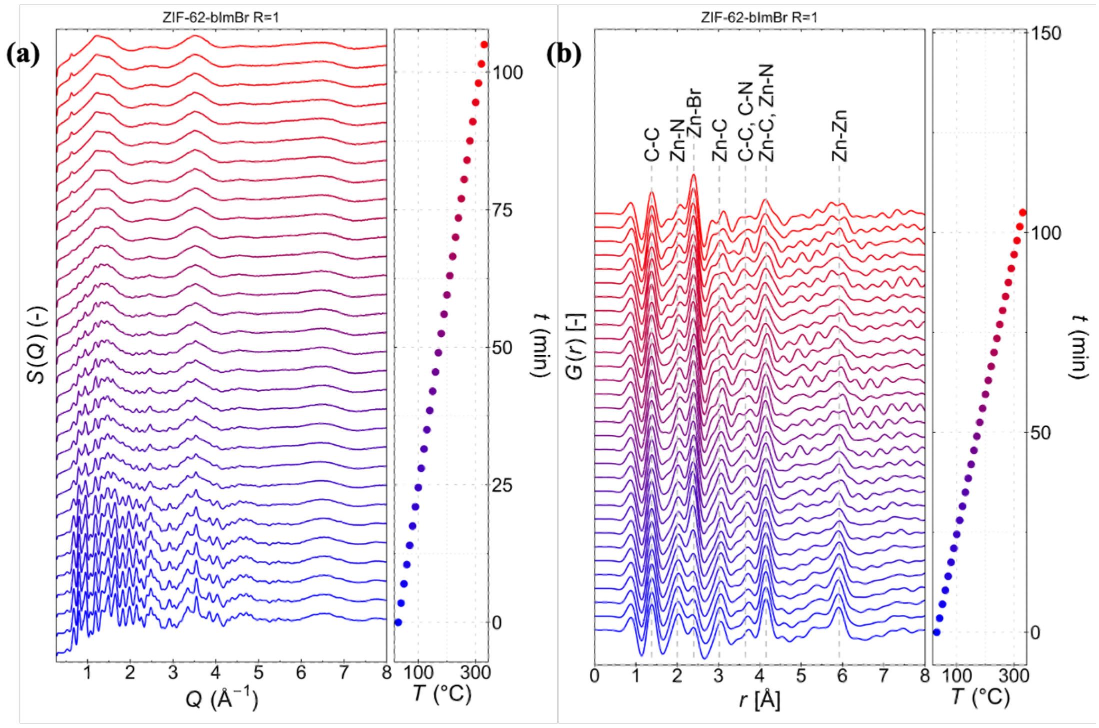

Supplementary Fig. 25. *In situ* X-ray total scanning variable temperature data for ZIF-62-bImBr *R*=1: (a) structure factor *S*(*Q*) and (b) pair-distribution function *G*(*r*). The measurements start from the initially mixed crystals of ZIF and salt (*t*=0). The loss of Bragg peaks is noticed at around 280 °C. Source data are provided as a Source Data file.

Supplementary Fig. 26

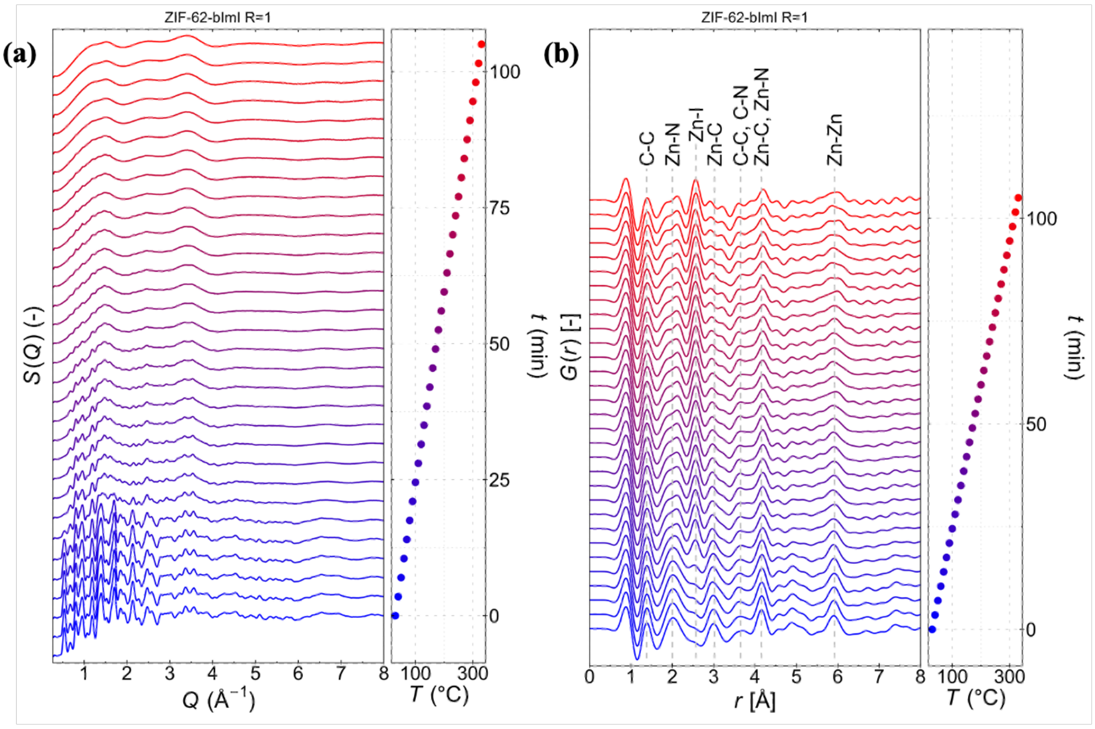

Supplementary Fig. 26. *In situ* X-ray total scanning variable temperature data for ZIF-62-bImI R=1: (a) structure factor $S(Q)$ and (b) pair-distribution function $G(r)$. The measurements start from the initially mixed crystals of ZIF and salt (t=0). The loss of Bragg peaks is noticed at around 290 °C. Source data are provided as a Source Data file.

Supplementary Fig. 27

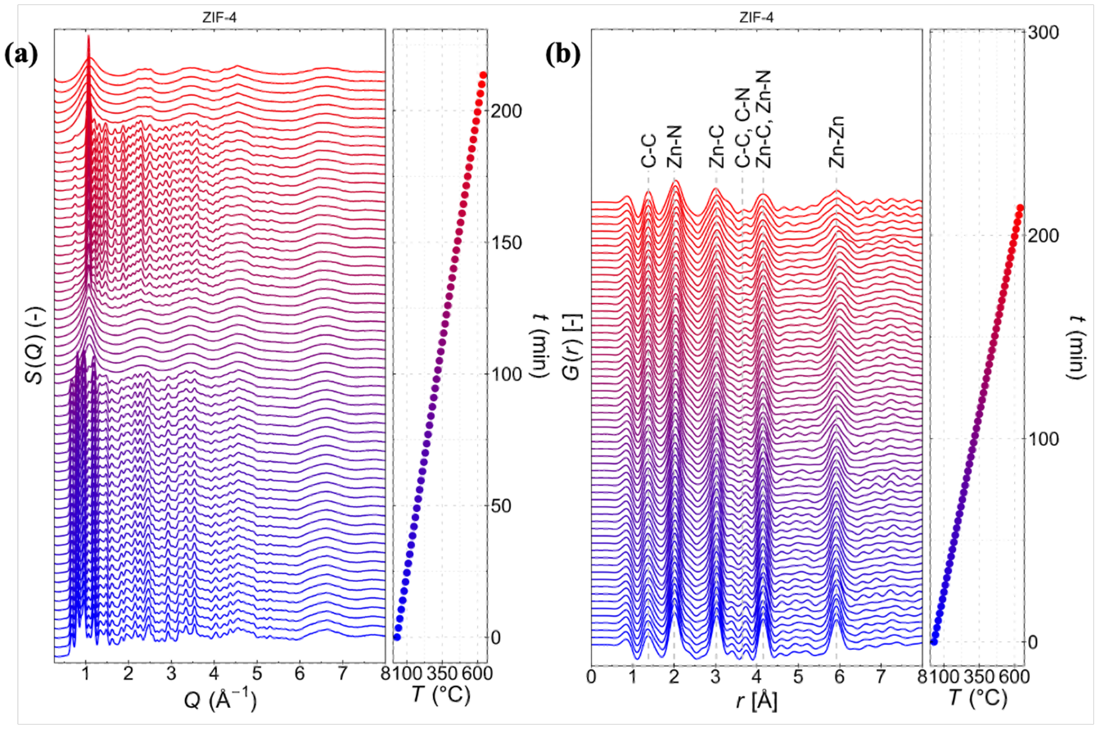

Supplementary Fig. 27. *In situ* X-ray total scanning variable temperature data for ZIF-4: (a) structure factor $S(Q)$ and (b) pair-distribution function $G(r)$. The measurements start from the crystal (t=0). The loss of Bragg peaks is noticed at around 300-310 °C and 590 °C, while recrystallization from a high-density amorphous ZIF phase into ZIF-zni is found to appear around ~370°C. Source data are provided as a Source Data file.

Supplementary Fig. 28

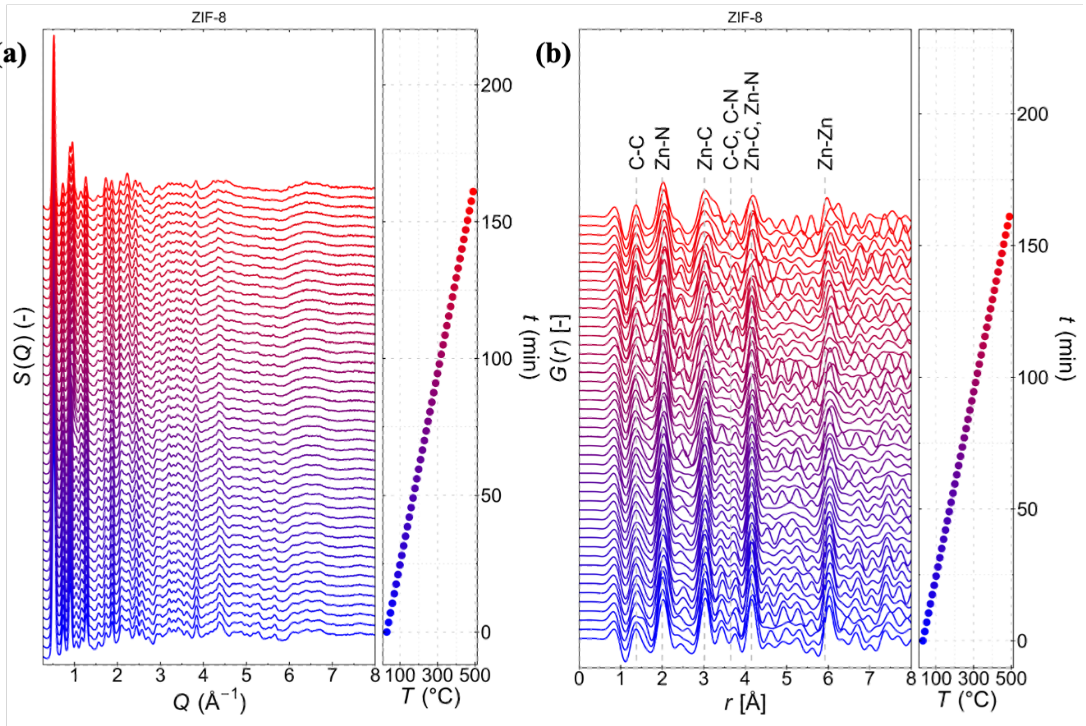

Supplementary Fig. 28. *In situ* X-ray total scanning variable temperature data for ZIF-8: (a) structure factor $S(Q)$ and (b) pair-distribution function $G(r)$. The measurements start from the crystal (t=0). No melting is observed. Source data are provided as a Source Data file.

Supplementary Fig. 29

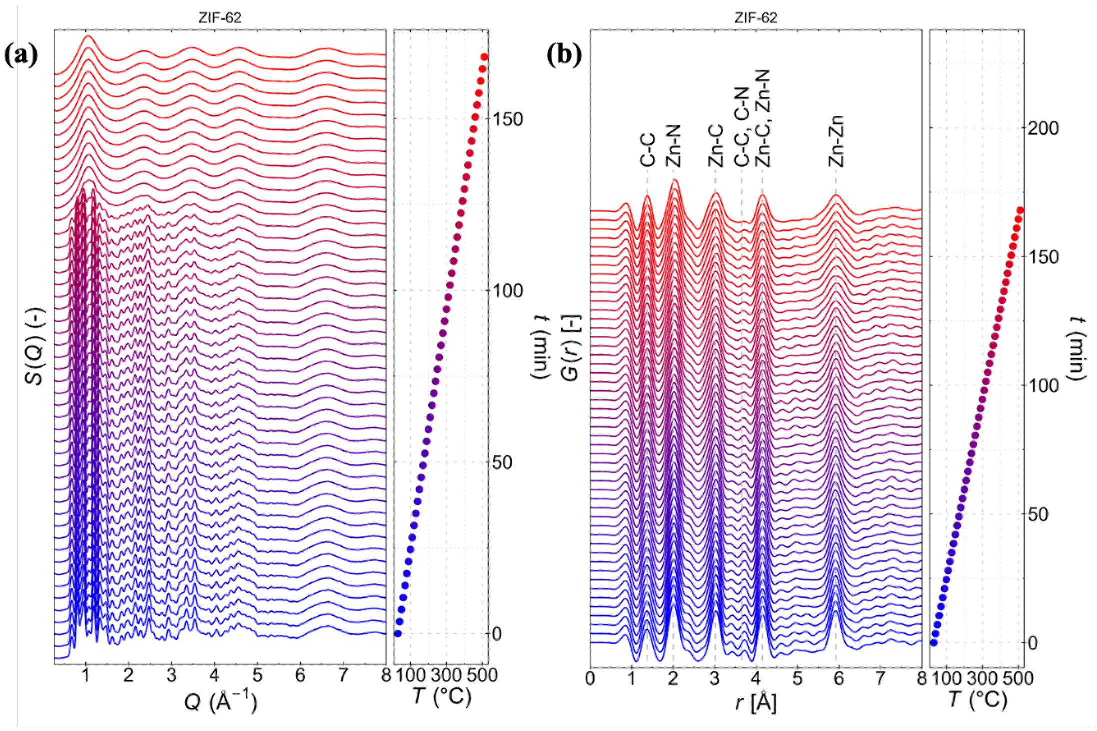

Supplementary Fig. 29. *In situ* X-ray total scanning variable temperature data for ZIF-62: (a) structure factor $S(Q)$ and (b) pair-distribution function $G(r)$. The measurements start from the crystal (t=0). The loss of Bragg peaks is noticed at around 400 °C. Source data are provided as a Source Data file.

Supplementary Fig. 30

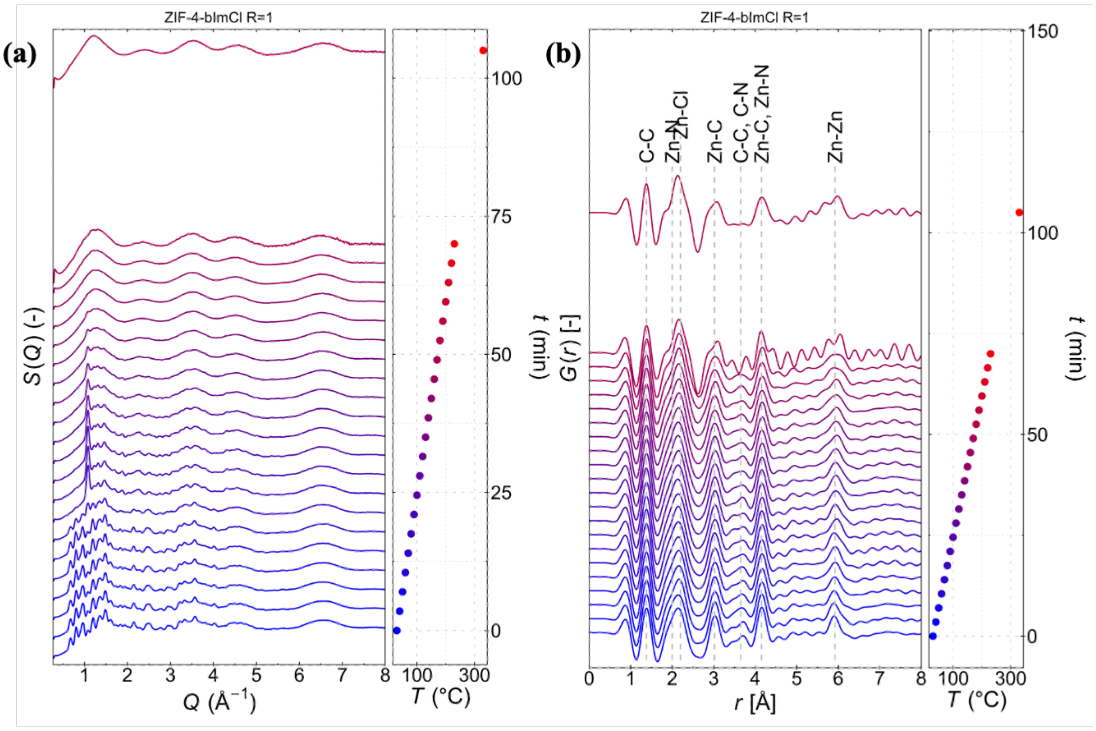

Supplementary Fig. 30. *In situ* X-ray total scanning variable temperature data for ZIF-4-bImCl R=1: (a) structure factor $S(Q)$ and (b) pair-distribution function $G(r)$. The measurements start from the initially mixed crystals of ZIF and salt (t=0). The loss of Bragg peaks is noticed at around 200 °C. The lack of spectra indicates that the beam signal was lost, either due to beam dump or because the ZIF liquid moved out of the X-ray beam during measurements. Source data are provided as a Source Data file.

Supplementary Fig. 31

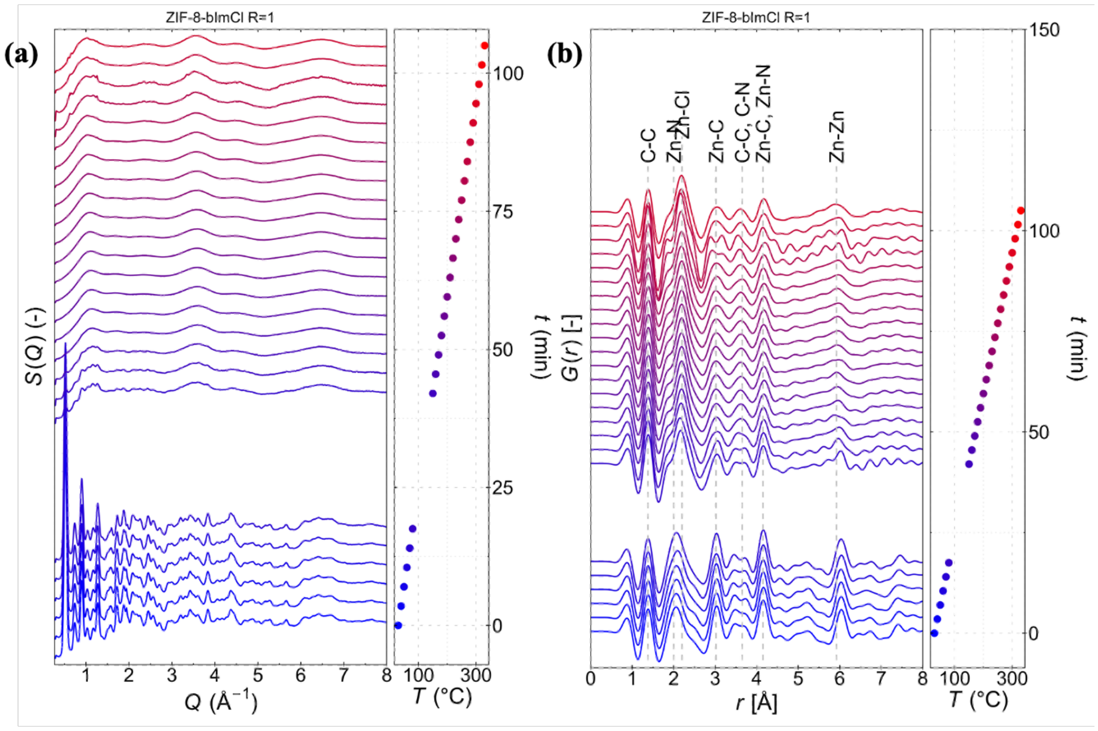

Supplementary Fig. 31. *In situ* X-ray total scanning variable temperature data for ZIF-8-bImCl R=1: (a) structure factor $S(Q)$ and (b) pair-distribution function $G(r)$. The measurements start from the initially mixed crystals of ZIF and salt (t=0). The loss of Bragg peaks is noticed at around 180 °C. The lack of spectra indicates that the beam signal was lost, either due to beam dump or because the ZIF liquid moved out of the X-ray beam during measurements. Source data are provided as a Source Data file.

Supplementary Fig. 32

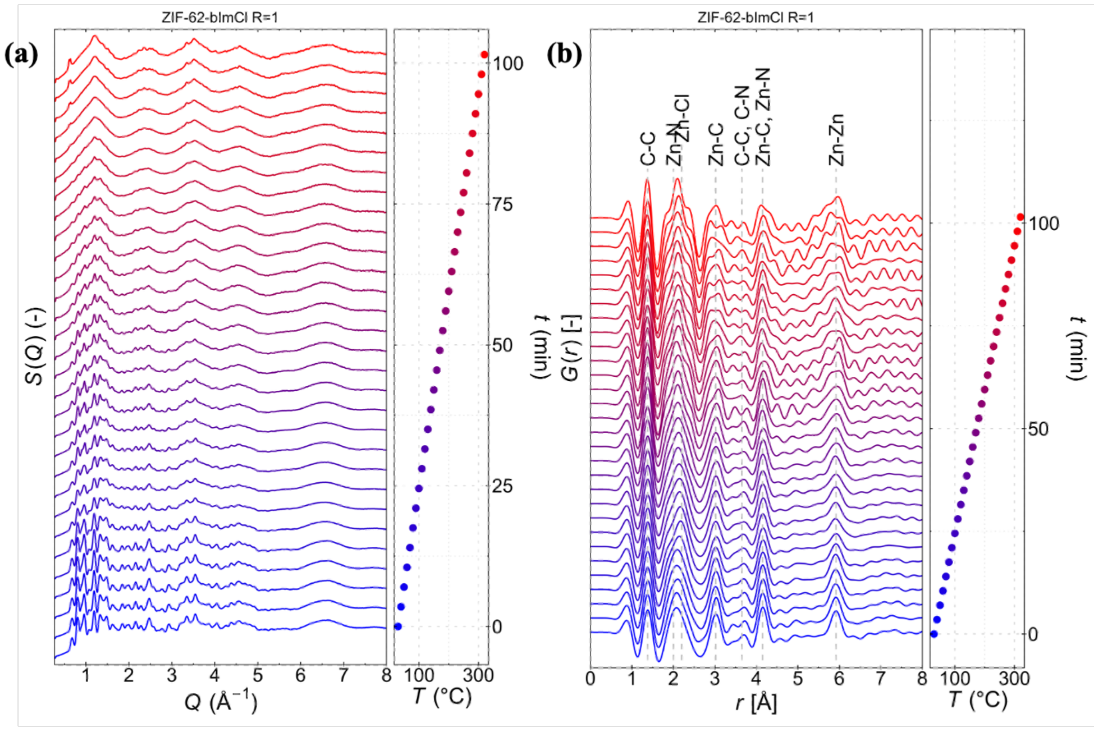

Supplementary Fig. 32. *In situ* X-ray total scanning variable temperature data for ZIF-62-bImCl *R*=1: (a) structure factor *S*(*Q*) and (b) pair-distribution function *G*(*r*). The measurements start from the initially mixed crystals of ZIF and salt (*t*=0). The loss of Bragg peaks is noticed at around 300 °C. Source data are provided as a Source Data file.

Supplementary Fig. 33

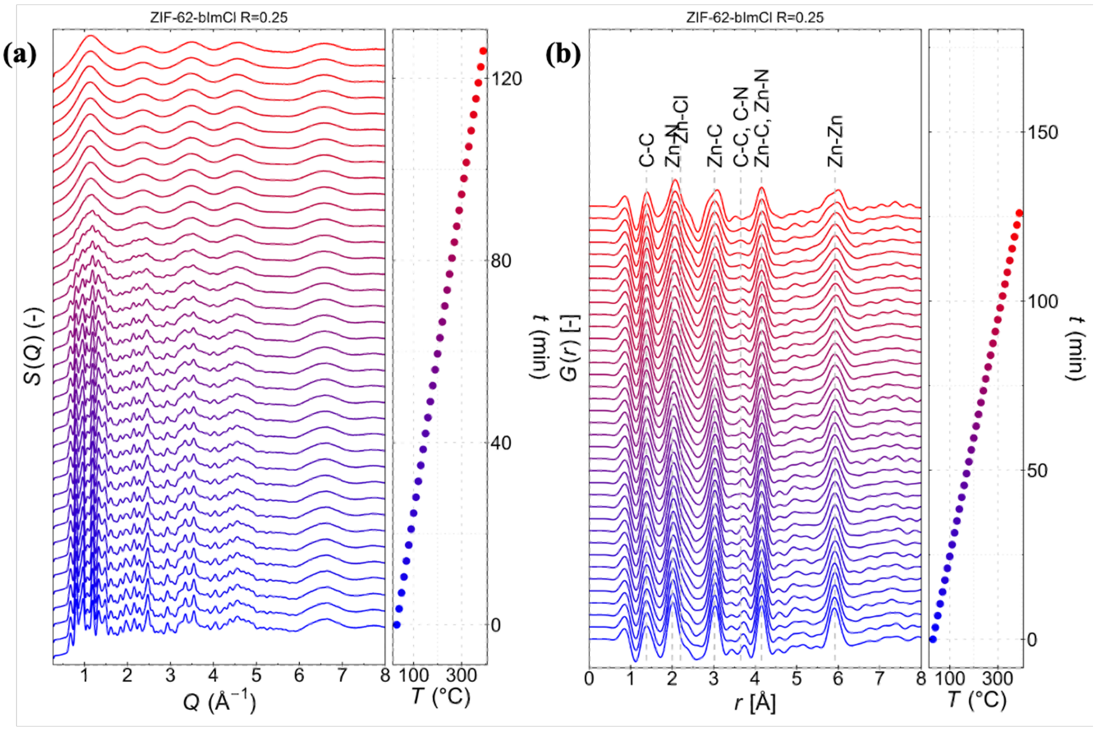

Supplementary Fig. 33. *In situ* X-ray total scanning variable temperature data for ZIF-62-bImCl R=0.25: (a) structure factor $S(Q)$ and (b) pair-distribution function $G(r)$. The measurements start from the initially mixed crystals of ZIF and salt (t=0). The loss of Bragg peaks is noticed at around 290 °C. Source data are provided as a Source Data file.

Supplementary Fig. 34

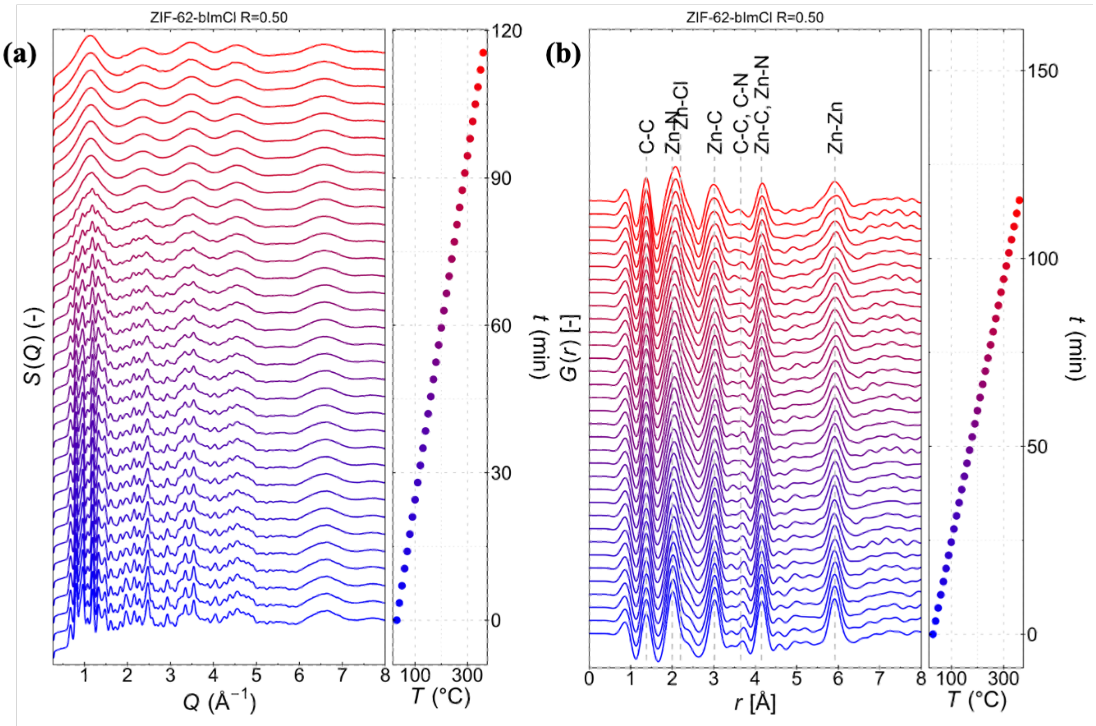

Supplementary Fig. 34. *In situ* X-ray total scanning variable temperature data for ZIF-62-bImCl R=0.5: (a) structure factor $S(Q)$ and (b) pair-distribution function $G(r)$. The measurements start from the initially mixed crystals of ZIF and salt (t=0). The loss of Bragg peaks is noticed at around 280 °C. Source data are provided as a Source Data file.

Supplementary Fig. 35

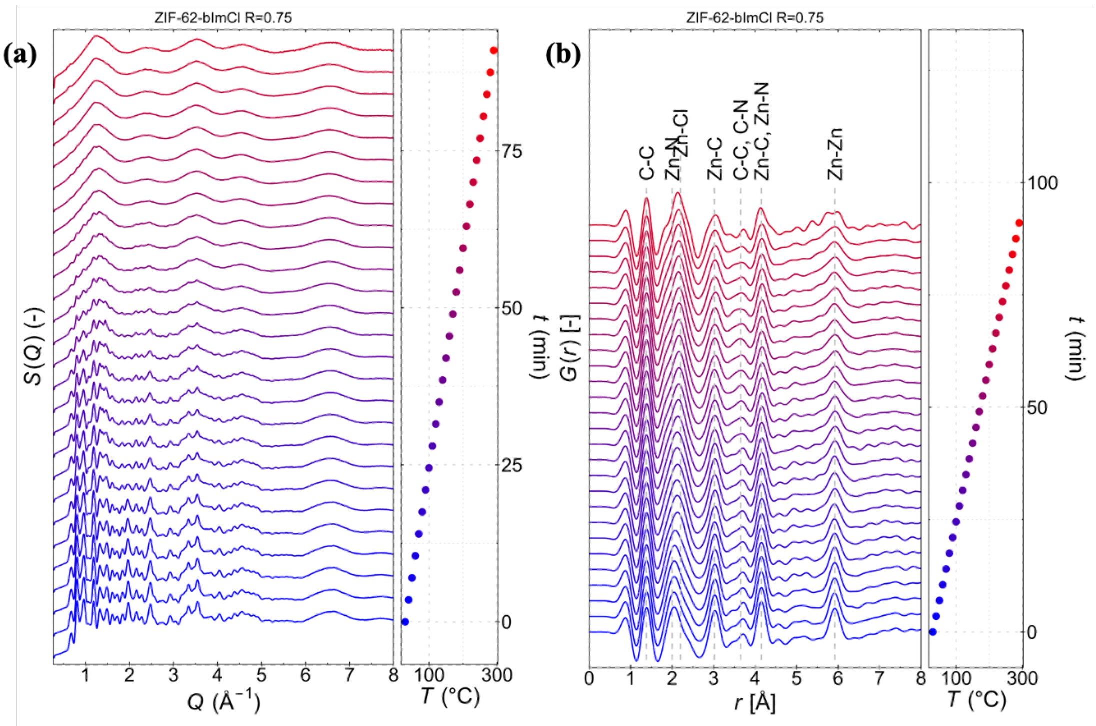

Supplementary Fig. 35. *In situ* X-ray total scanning variable temperature data for ZIF-62-bImCl R=0.75: (a) structure factor $S(Q)$ and (b) pair-distribution function $G(r)$. The measurements start from the initially mixed crystals of ZIF and salt (t=0). The loss of Bragg peaks is noticed at around 250 °C. Source data are provided as a Source Data file.

Supplementary Fig. 36

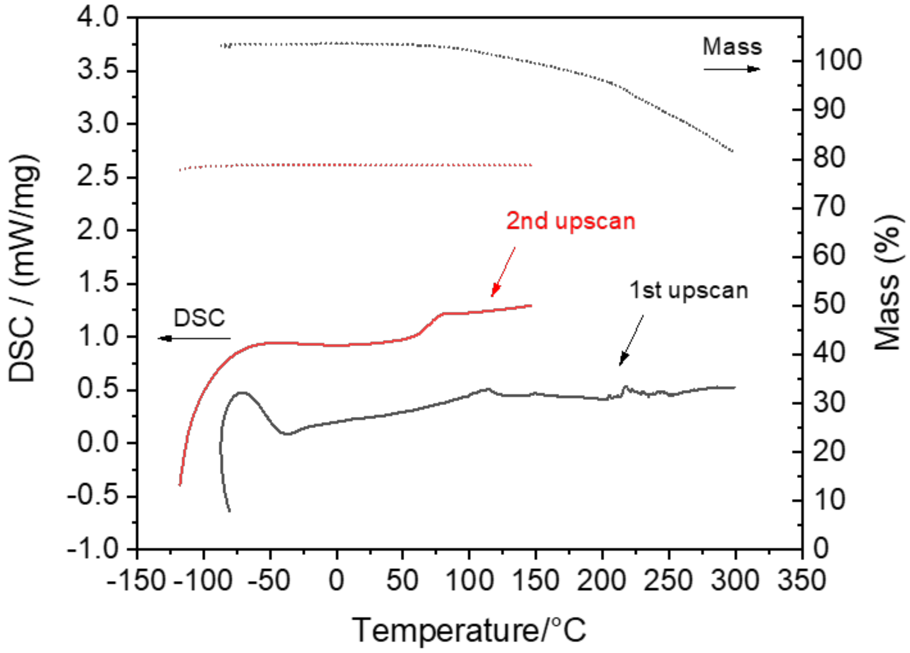

Supplementary Fig. 36. First and second DSC (solid lines) and TGA (dotted lines) heating upscans at 10 K min^{-1} in Al crucible (with a pinhole on the lid) of ZIF-62 modified with H_2bImCl at R = 1.0. Source data are provided as a Source Data file.

Supplementary Fig. 37

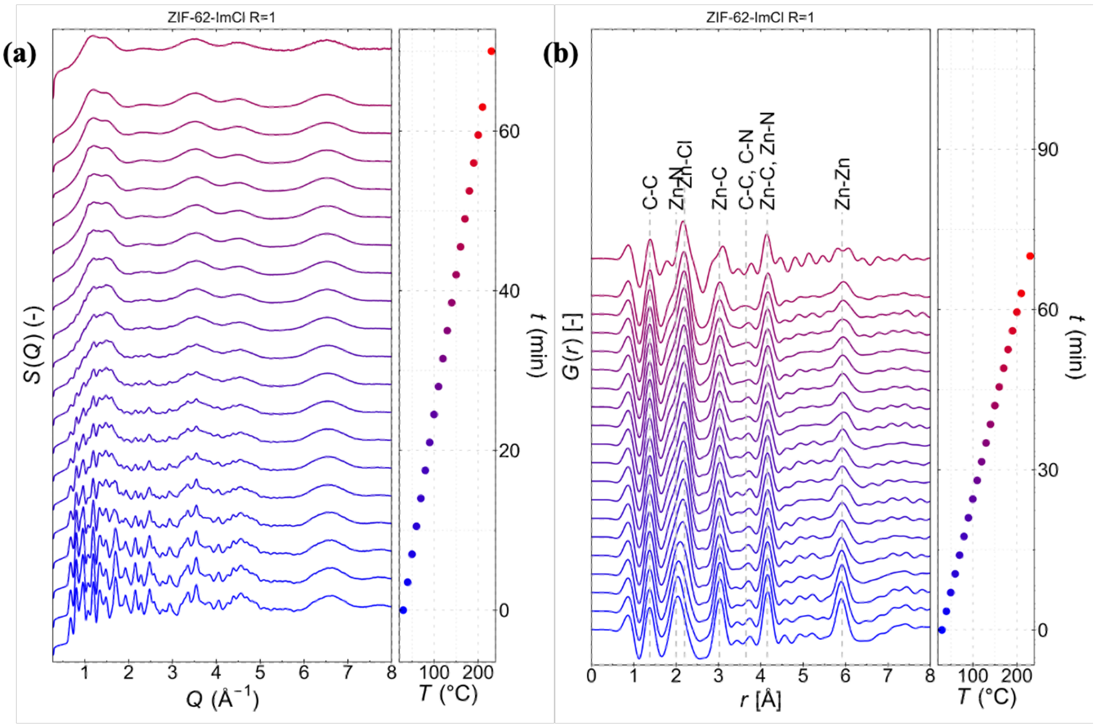

Supplementary Fig. 37. *In situ* X-ray total scanning variable temperature data for ZIF-62-ImCl R=1: (a) structure factor $S(Q)$ and (b) pair-distribution function $G(r)$. The measurements start from the initially mixed crystals of ZIF and salt (t=0). The loss of Bragg peaks is noticed at around 200 °C. The lack of spectra indicates that the beam signal was lost, either due to beam dump or because the ZIF liquid moved out of the X-ray beam during measurements. Source data are provided as a Source Data file.

Supplementary Fig. 38

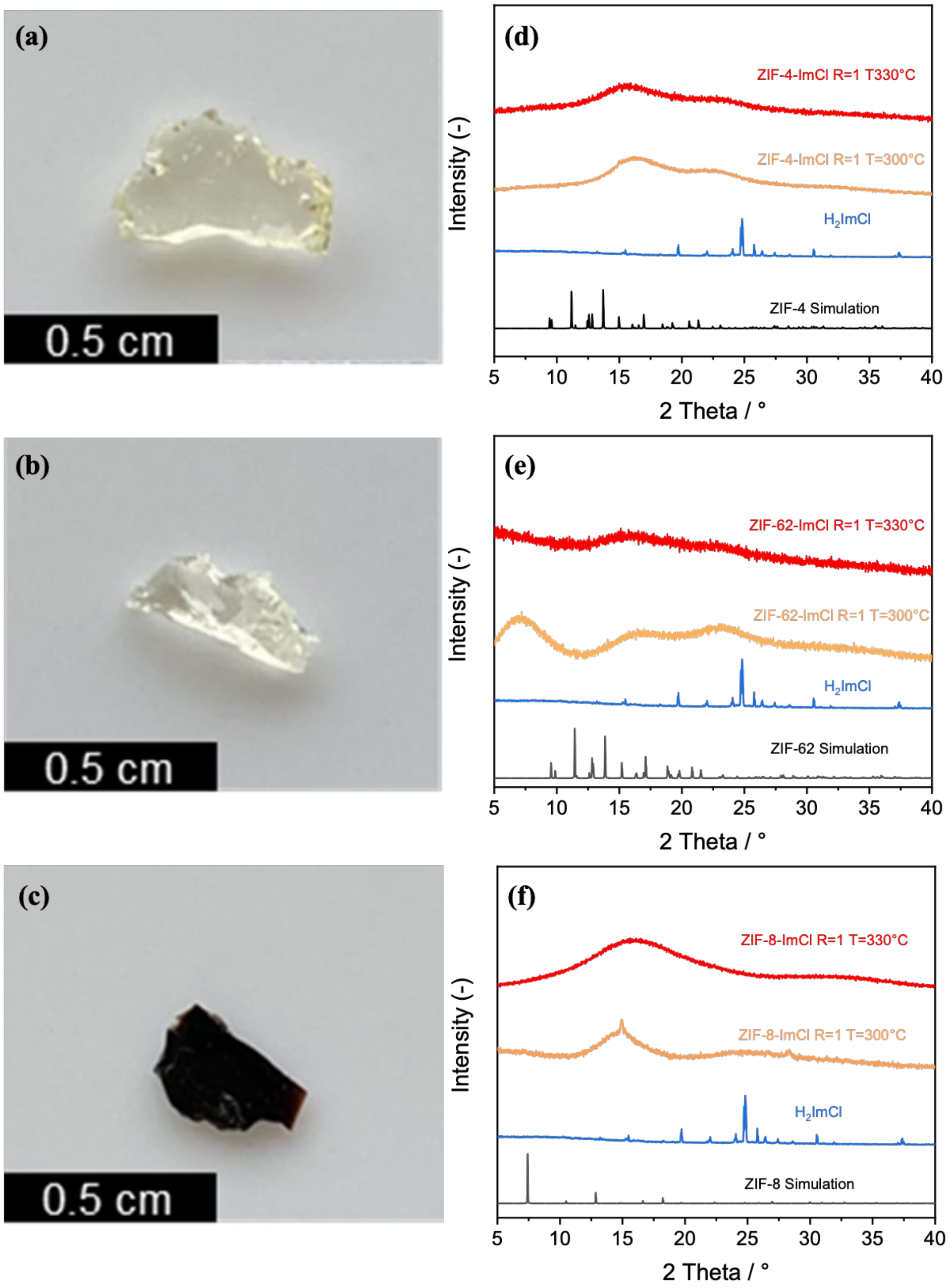

Supplementary Fig. 38. (a-c) Images of (a) ZIF-4-ImCl *R*=1, (b) ZIF-62-ImCl *R*=1, and (c) ZIF-8-ImCl *R*=1 glasses after melt-quenching from 300 °C. (d-f) X-ray diffraction patterns of (d) ZIF-4-ImCl *R*=1, (e) ZIF-62-ImCl *R*=1, and (f) ZIF-8-ImCl *R*=1 glasses prepared at different temperatures as well as simulated XRD spectra of the pure ZIF-4, ZIF-8, and ZIF-62 crystals (CCDC deposition numbers: 602538, 1429243, and 671070, respectively). Source data are provided as a Source Data file.

Supplementary Fig. 39

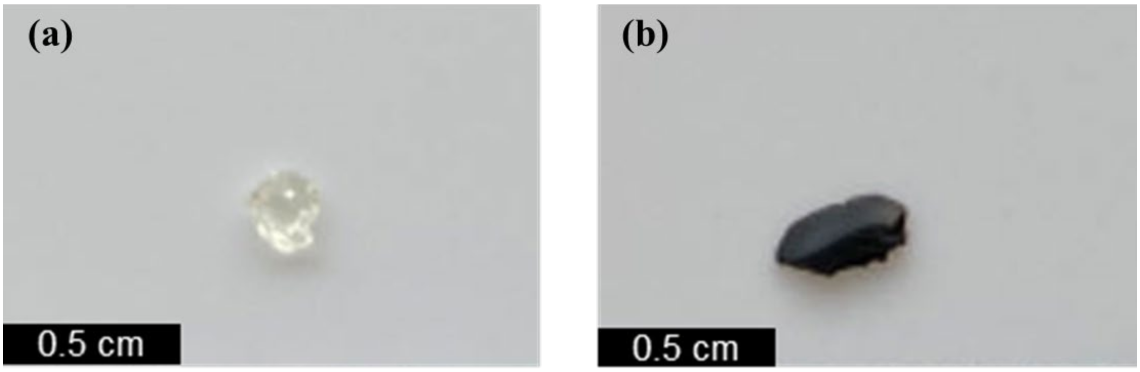

Supplementary Fig. 39. Images of (a) ZIF-62-PyCl R=1 and (b) ZIF-8-PyCl R=1 glass samples formed by melt-quenching from 300 °C.

Supplementary Fig. 40

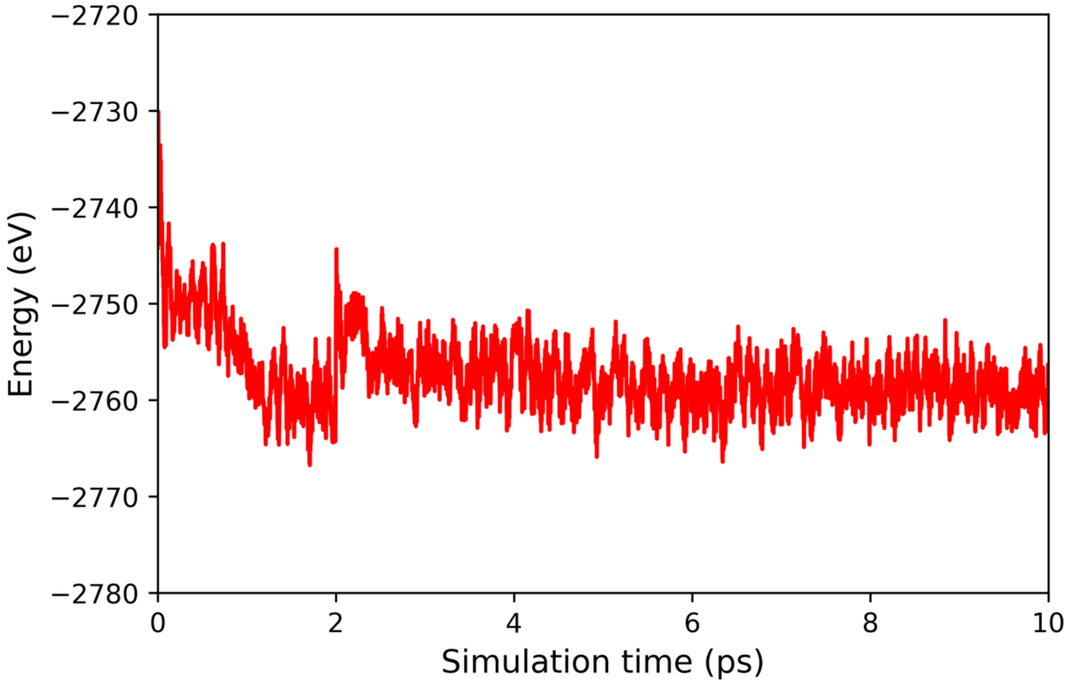

Supplementary Fig. 40. Energy during 10 ps of *ab initio* molecular dynamics simulation of the ZIF-62-bImCl *R*=0.5 system. The sample was prepared by using a single unit cell of ZIF-62 where 8 units of H_2bImCl were added. The system was equilibrated to 1000 K during the simulation run. Initial structural relaxation was performed prior to the presented simulation. Source data are provided as a Source Data file.

Supplementary Fig. 41

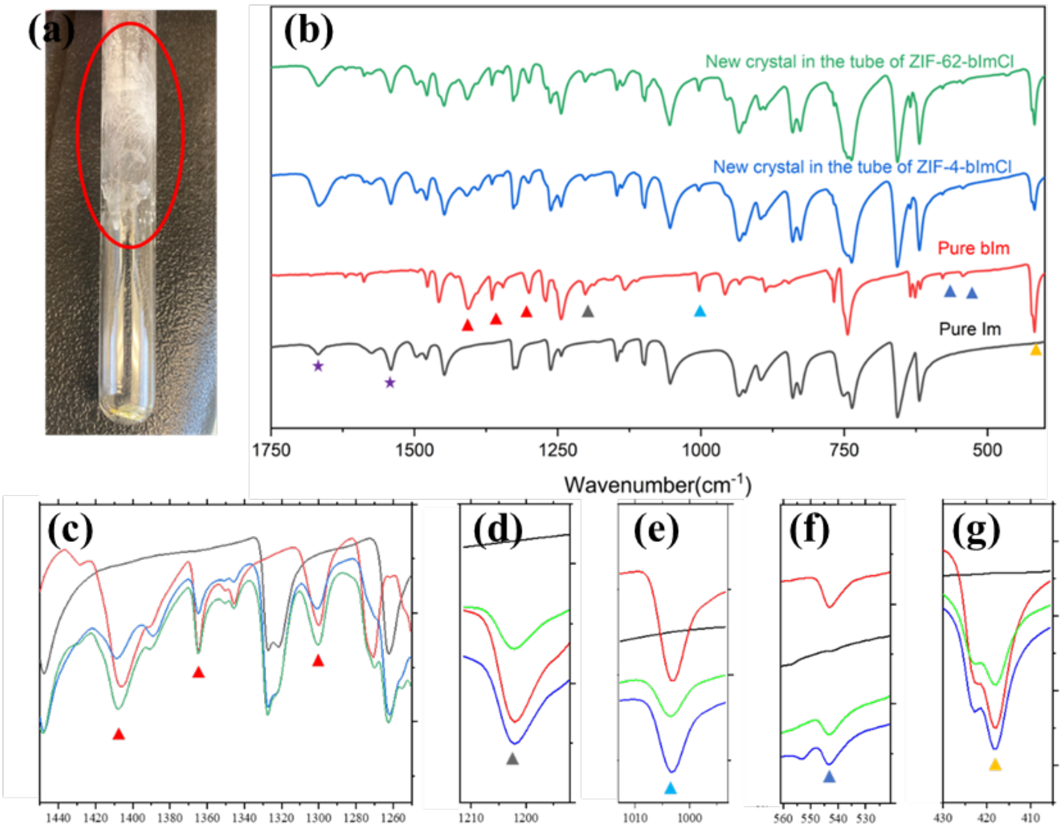

Supplementary Fig. 41. (a) Image of crystals formed at the top part of the tube for ZIF-62-bImCl *R*=1 upon heating to 300 °C. (b) FT-IR spectra of white condensed powder, showing clear characteristics of imidazole (HIm) and benzimidazole (HbIm). (c)-(g) Enlarged views of the data in panel (b). Source data are provided as a Source Data file.

Supplementary Fig. 42

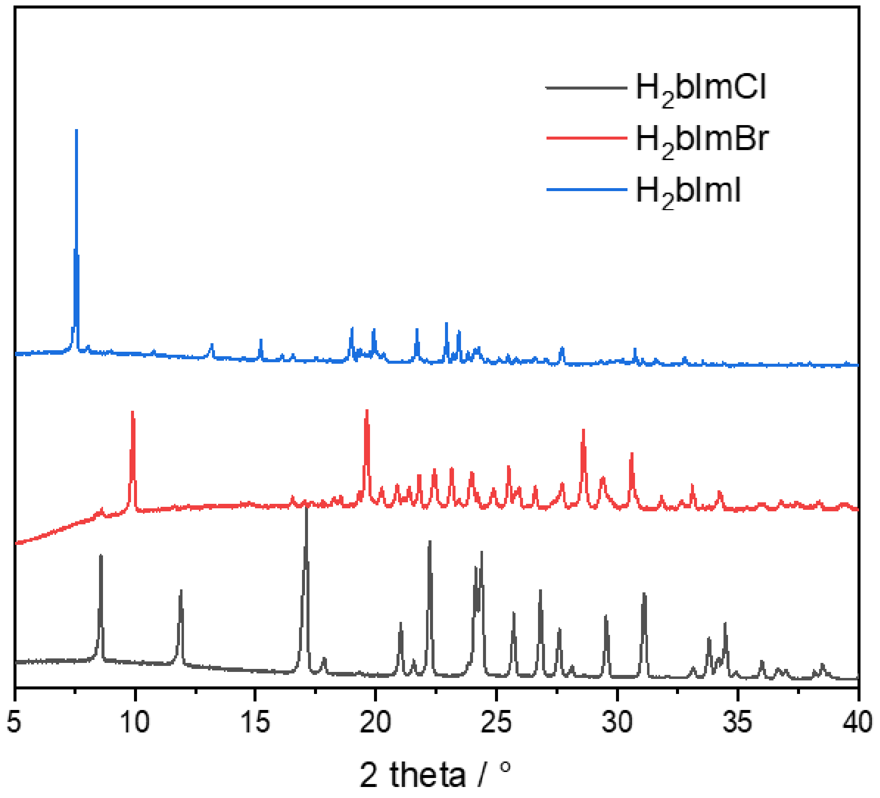

Supplementary Fig. 42. X-ray diffraction patterns of benzimidazolium chloride (black line), benzimidazolium bromide (red line), and benzimidazolium iodide (blue line). Patterns are measured using Cu Kα radiation (λ = 1.5406 Å). Source data are provided as a Source Data file.

Supplementary Fig. 43

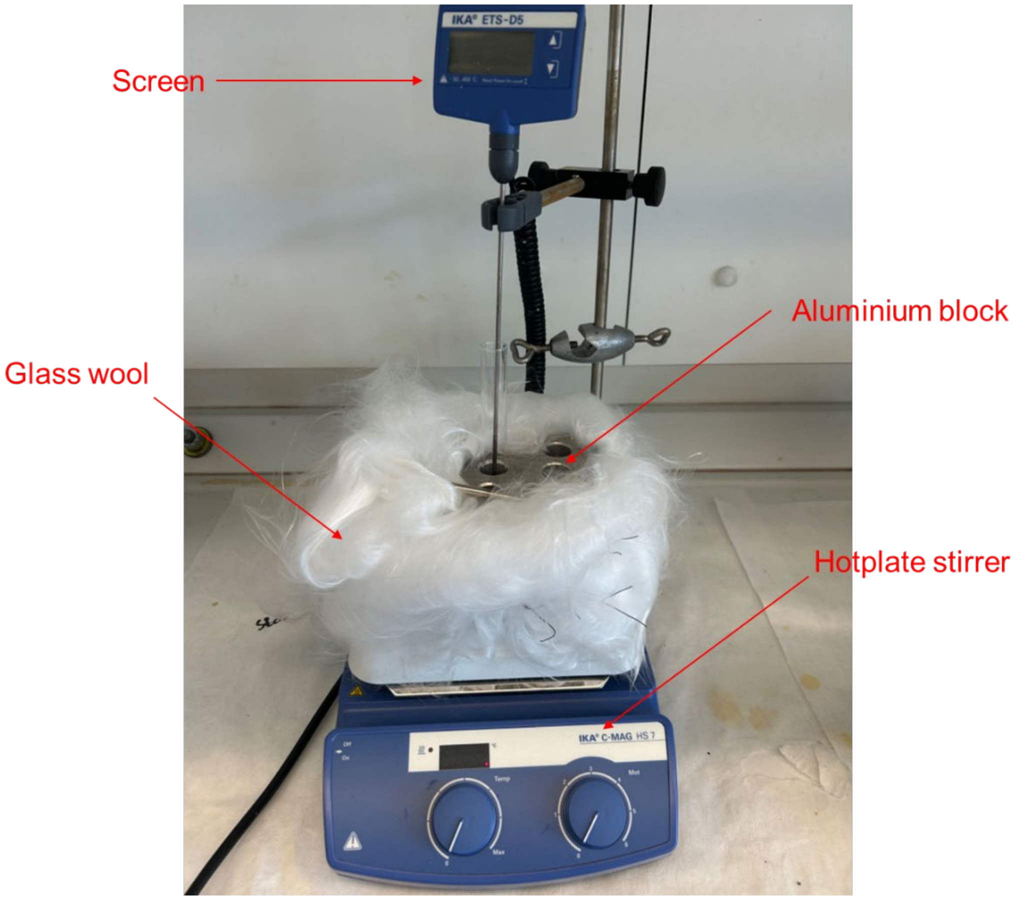

Supplementary Fig. 43. Employed heating setup to prepare all modified glasses in this work, consisting of an aluminum block on top of an IKA C-MAG HS7 hotplate stirrer capable of reaching a temperature of ~450 °C.

Supplementary Fig. 44

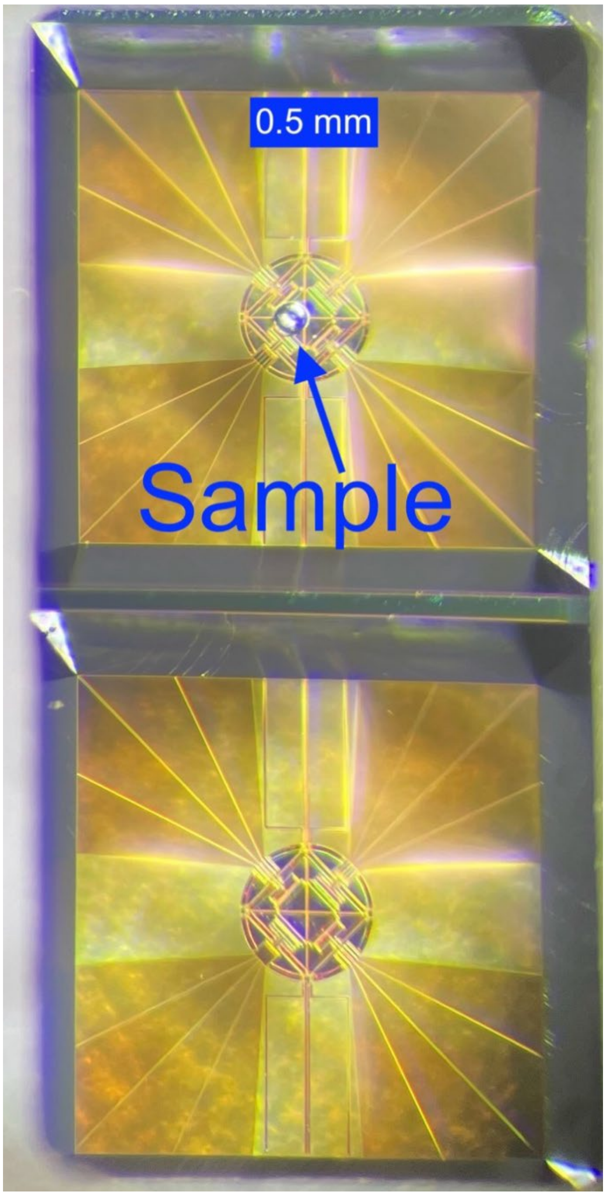

Supplementary Fig. 44. Sample from fast scanning calorimetry measurements shown loaded onto the calorimeter chip. The sample was premelted onto the chip and is around 0.15 mm in diameter.

Supplementary Fig. 45

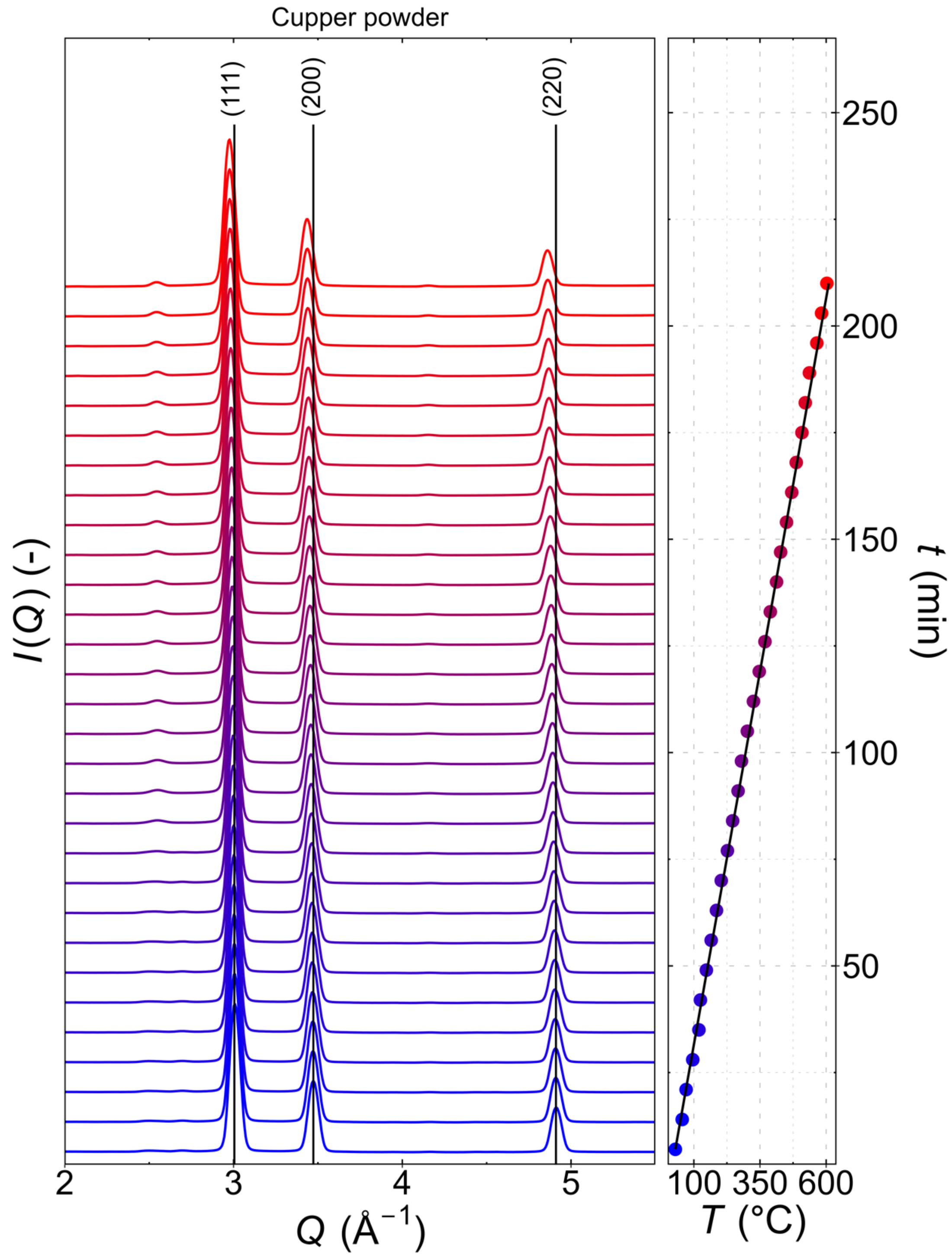

Supplementary Fig. 45. *In situ* x-ray total scattering intensity, *I*(*Q*) of copper powder as a function of temperature from room temperature to 600 °C used for the temperature calibration of the synchrotron setup. Source data are provided as a Source Data file.

Supplementary Fig. 46

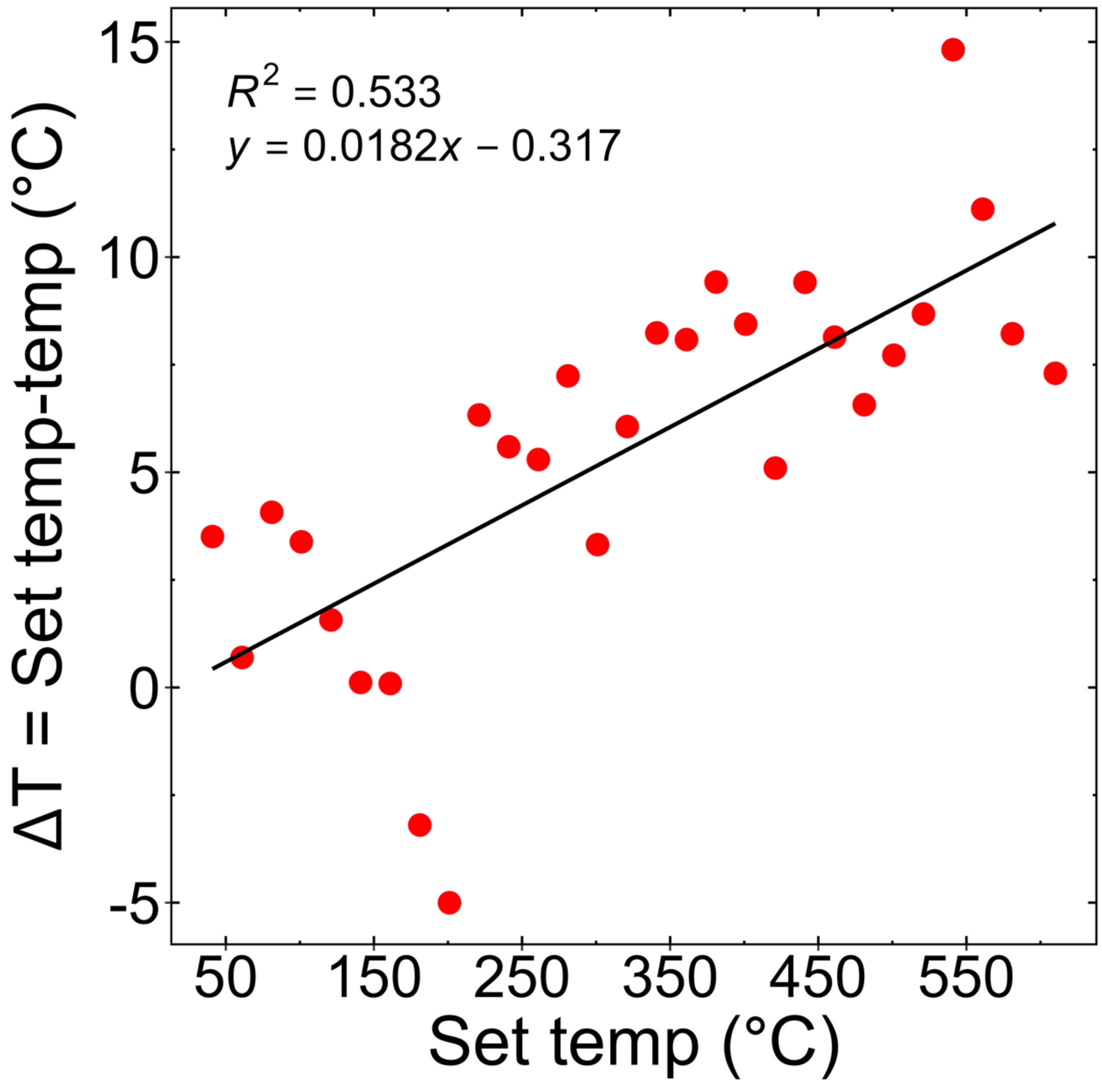

Supplementary Fig. 46. Difference in temperature between temperature set point and temperature ($\Delta T = T_{set} - T$) as a function of the set temperature. The difference is determined from the thermal expansion of the (2 0 0) lattice parameter of Cu. Source data are provided as a Source Data file.

Supplementary Fig. 47

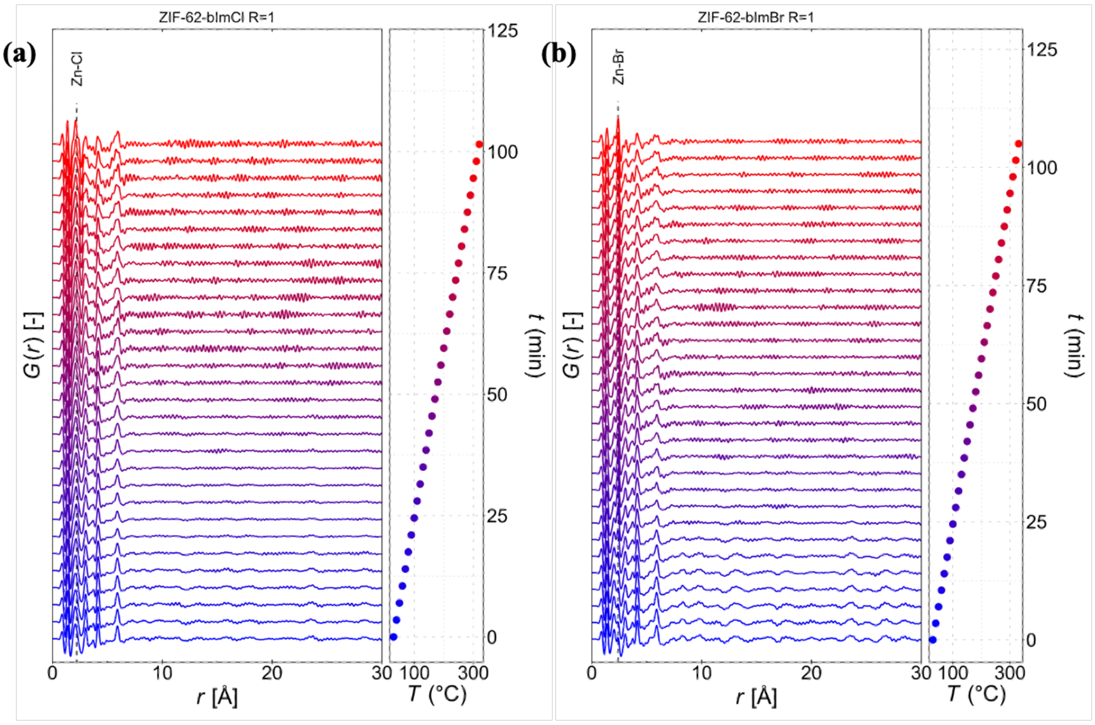

Supplementary Fig. 47 Pair-distribution function $G(r)$ variable temperature data for (a) ZIF-62-bImCl R=1 (b) and ZIF-62-bImBr R=1, showing data up to r=30 Å. We note how this is the same data sets as presented in Supplementary Figs. 30 and 34 but shown in a different real-space range. No significant crystallization is apparent in the long-range interactions, but the signal is slightly obscured due to little sample in the beam and therefore low-quality data at high Q, effectively inducing a noisy Fourier transform. The most prominent peaks are, however, still noticeable, such as the Zn-Cl and Zn-Br interactions in panels (a) and (b), respectively. Source data are provided as a Source Data file.

Supplementary References

1. Zheng Q*, et al.* Understanding Glass through Differential Scanning Calorimetry. *Chemical Reviews* **119**, 7848-7939 (2019).

2. Ordoñez MJC, Balkus KJ, Ferraris JP, Musselman IH. Molecular sieving realized with ZIF-8/Matrimid® mixed-matrix membranes. *Journal of Membrane Science* **361**, 28-37 (2010).

3. Jain P*, et al.* Raman Evidence of Multiple Adsorption Sites and Structural Transformation in ZIF-4. *Inorganic Chemistry* **62**, 7703-7715 (2023).

4. Bennett TD*, et al.* Hybrid glasses from strong and fragile metal-organic framework liquids. *Nat Commun* **6**, 8079 (2015).

5. Qiao A*, et al.* A metal-organic framework with ultrahigh glass-forming ability. *Science Advances* **4**, eaao6827 (2018).

6. Sørensen SS*, et al.* Water as a Modifier in a Hybrid Coordination Network Glass. *Small* **19**, 2205988 (2023).

7. Nieto CI, Cabildo P, García MÁ, Claramunt RM, Alkorta I, Elguero J. An experimental and theoretical NMR study of NH-benzimidazoles in solution and in the solid state: proton transfer and tautomerism. *Beilstein Journal of Organic Chemistry* **10**, 1620-1629 (2014).

8. Ueda T, Nagatomo S, Masui H, Nakamura N, Hayashi S. Hydrogen Bonds in Crystalline Imidazoles Studied by 15N NMR and ab initio MO Calculations. *Zeitschrift für Naturforschung A* **54**, 437-442 (1999).

9. Song X-j, McDermott AE. Proton transfer dynamics and N—H bond lengthening in N—H···N model systems: a solid-state NMR study. *Magnetic Resonance in Chemistry* **39**, S37-S43 (2001).

10. Bouchmella K, Dutremez SG, Alonso B, Mauri F, Gervais C. 1H, 13C, and 15N Solid-State NMR Studies of Imidazole- and Morpholine-Based Model Compounds Possessing Halogen and Hydrogen Bonding Capabilities. *Crystal Growth & Design* **8**, 3941-3950 (2008).
